# TRP Channels and Small GTPases Interplay in the Main Hallmarks of Metastatic Cancer

**DOI:** 10.3389/fphar.2020.581455

**Published:** 2020-09-29

**Authors:** Giorgia Chinigò, Alessandra Fiorio Pla, Dimitra Gkika

**Affiliations:** ^1^Laboratory of Cellular and Molecular Angiogenesis, Department of Life Sciences and Systems Biology, University of Torino, Torino, Italy; ^2^Laboratoire de Cell Physiology, Université de Lille, Department of Life Sciences, Univ. Lille, Inserm, U1003—PHYCEL, Lille, France; ^3^Univ. Lille, CNRS, INSERM, CHU Lille, Centre Oscar Lambret, UMR 9020-UMR 1277-Canther-Cancer Heterogeneity, Plasticity and Resistance to Therapies, Lille, France; ^4^Institut Universitaire de France (IUF), Paris, France

**Keywords:** transient receptor potential channels, small guanosine triphosphatases, cancer hallmarks, metastasis, molecular signaling, migration, invasion, tumor vascularization

## Abstract

Transient Receptor Potential (TRP) cations channels, as key regulators of intracellular calcium homeostasis, play a central role in the essential hallmarks of cancer. Among the multiple pathways in which TRPs may be involved, here we focus our attention on the ones involving small guanosine triphosphatases (GTPases), summarizing the main processes associated with the metastatic cascade, such as migration, invasion and tumor vascularization. In the last decade, several studies have highlighted a bidirectional interplay between TRPs and small GTPases in cancer progression: TRP channels may affect small GTPases activity *via* both Ca^2+^-dependent or Ca^2+^-independent pathways, and, conversely, some small GTPases may affect TRP channels activity through the regulation of their intracellular trafficking to the plasma membrane or acting directly on channel gating. In particular, we will describe the interplay between TRPC1, TRPC5, TRPC6, TRPM4, TRPM7 or TRPV4, and Rho-like GTPases in regulating cell migration, the cooperation of TRPM2 and TRPV2 with Rho GTPases in increasing cell invasiveness and finally, the crosstalk between TRPC1, TRPC6, TRPM8, TRPV4 and both Rho- and Ras-like GTPases in inducing aberrant tumor vascularization.

## Introduction

Ca^2+^ signaling plays a central role in the regulation of many important cellular functions and indeed, not surprisingly, a dysregulation of Ca^2+^ homeostasis has been observed in various pathological conditions, including tumorigenesis (Monteith et al., 2017). Changes in Ca^2+^ signaling leading to dysfunctions in cancer cells are due to alterations in the so-called “Ca^2+^-signaling toolkit” (Berridge et al., 2003) which includes, among others, ion channels, responsible for altered fluxes within the cell, and several Ca^2+^-dependent effectors, which mediate various signal transduction pathways in response to altered Ca^2+^ homeostasis.

In recent years, the involvement of Ca^2+^-permeable channels in neoplastic diseases has been extensively investigated and a direct correlation between their dysregulation and cancer development has been shown (Monteith et al., 2017). Among them, Transient Receptor Potential (TRP) channels have revealed an important involvement in the regulation of many signaling pathways associated with tumor progression (Yang and Kim, 2020). One of the key features of TRP channels is their polymodal activation mechanism and their involvement in different signal transduction pathways. Among the multiple pathways TRPs are signaling through, the ones involving specific intracellular messengers belonging to the family of small guanosine triphosphatases (GTPases) are emerging as essential in tumorigenesis in the last decade. Therefore, in this review, we will focus on the interplay between TRPs and small GTPases, and its role in several aspects of the metastatic process (Aspenström, 2004; Clayton and Ridley, 2020).

The metastatic cascade leads to spread of malignant cells from the primary tumor through the lymph or blood circulation to establish secondary growth in a distant organ; it is a multistep process involving highly complex structural and functional alterations within cancer cells. Primary tumor cells are primed for dissemination by the process of epithelial-mesenchymal transition (EMT), during which they assume a more aggressive mesenchymal-like phenotype. This phenotypic switch allows them to detach from the primary tumor mass and to acquire migratory and invasive properties, in order to move from their original location, migrate and invade the extracellular matrix (ECM) and endothelium to spread to secondary sites and form metastases (Roche, 2018). All the steps involved in metastatic cascade (EMT, cell migration, invasion and tumor vascularization) are regulated by the intracellular Ca^2+^ concentration (Berridge et al., 2003) and specifically by the TRP-mediated calcium influx (Fels et al., 2018), as well as by the most important small GTPases (Rho-like and Ras-like) involved in cytoskeletal dynamics and cell polarity (Ungefroren et al., 2018). Here, we discuss TRPs and small GTPases contribution in cancer progression, focusing on signaling pathways involving a direct interplay between TRPs and small GTPases in three main metastatic cancer hallmarks: migration, invasion and tumor vascularization.

### TRP Channels

TRP channels mainly act as signal transducers by altering membrane potential or intracellular Ca^2+^ concentration in response to various environmental stimuli, including physical-chemical stimuli, such as temperature, pH changes, osmolarity, and pressure as well as endogenous and exogenous ligands (Nilius and Owsianik, 2011). TRP channels have been shown to play a central role in carcinogenesis as well as in various late stages of tumor progression. In particular, it has been shown that changes in the expression of TRP channels are correlated with the progression of different types of cancer. To date, most changes involving TRP proteins do not involve mutations in the TRP gene but rather dysregulation of the wild-type TRP protein expression levels, depending on the stage of cancer (Gkika and Prevarskaya, 2011; Lehen’kyi and Prevarskaya, 2011; Bernardini et al., 2015; Shapovalov et al., 2016). Moreover, several recent studies have highlighted a direct correlation between cancer patient survival and TRP channel expression. Tumor differential expression of the main TRP channels discussed in this review and its correlation with patients’ survival are summarized in **Table 1**.

**Table 1:** TRPs expression in cancer and correlation with patient survival prognosis.

Channel	Cancer Type	Expression			Prognosis	References
		Healthy/Benign	Tumor	Invasive		
TRPC1	Breast basal/lymph nodes Breast ductal adenocarcinoma	Yes yes	↑ ↑	↑	poor	Azimi et al., 2017; Dhennin-duthille et al., 2011
TRPC5	Colon	yes	↑	↑	poor	Chen et al., 2017a
TRPC6	Prostate	yes	↑	↑		Yue et al., 2009
	Glioblastoma	yes	↑	↑		Chigurupati et al., 2010
	Esophageal squamous cell carcinoma	yes	↑	↑	poor	Zhang et al., 2013
	Breast/Invasive ductal adenocarcinoma	yes	↑			Guilbert et al., 2008; Dhennin-duthille et al., 2011
TRPM2	Breast	yes	↑	↑	Depends on subtype	Sumoza-Toledo et al., 2016
TRPM4	Prostate	yes	↑			Ashida et al., 2004; Singh et al., 2006
	Cervical	yes	↑			Narayan et al., 2007
TRPM7	Breast	yes	↑	↑	poor	Middelbeek et al., 2012; Meng et al., 2013
	Invasive ductal adenocarcinoma/lymph nodes	yes	↑	↑		Dhennin-duthille et al., 2011; Guilbert et al., 2013
	Pancreatic ductal adenocarcinoma/lymph nodes	yes	↑	↑	poor	Rybarczyk et al., 2012; Yee et al., 2015
	Nasopharyngeal	no	↑	↑	poor	Chen et al., 2015
	Ovarian; Prostate; Melanoma; Sarcoma			↑		Meng et al., 2013
TRPM8	Breast ductal adenocarcinoma	yes	↑	↑		Dhennin-duthille et al., 2011
	Prostate	yes	↑	loss		Tsavaler et al., 2001; Fuessel et al., 2003; Bidaux et al., 2005
	Urothelial carcinoma of bladder	yes	↑	↑	poor	Xiao et al., 2014
	Lung	Very low level	↑			Tsavaler et al., 2001
	Colon	no	↑			Tsavaler et al., 2001
	Melanoma	no	↑			Tsavaler et al., 2001
	Osteosarcoma	yes	↑	↑	poor	Zhao and Xu, 2016
TRPV2	Prostate	no		↑		Monet et al., 2010
	Bladder	yes (full isoform) (short isoform)	↑ ↓	↑ Loss		Caprodossi et al., 2008
	Esophageal squamous cell carcinoma	yes	↑	↑	poor	Zhou et al., 2014
	Gastric	yes	↑	↑	poor	Zoppoli et al., 2019
	Breast (Triple negative/basal subtype)	yes	↑		better	Elbaz et al., 2018
TRPV4	Breast	yes	↑	↑	poor	Lee et al., 2016; Lee et al., 2017
	Gastric		↑		poor	Lee et al., 2016
	Ovarian		↑		poor	Lee et al., 2016
	Glioma	yes	↑	↑	poor	Ou-yang et al., 2018

↑: increment; ↓: reduction.

These observations strongly indicate that TRPs play a significant role in cancer progression and more specifically in many processes underlying the metastatic cascade, making them promising candidates as both molecular biomarkers and therapeutic targets in various types of cancer (Gkika and Prevarskaya, 2011; Fiorio Pla and Gkika, 2013; Bernardini et al., 2015; Lastraioli et al., 2015; Litan and Langhans, 2015; Fels et al., 2018). It has been shown that TRP-mediated effects on metastatic cancer cell behavior are mainly associated to their Ca^2+^ permeability. Indeed, through the regulation of intracellular Ca^2+^ concentration, both in the cytosol and within subcellular organelles, TRP channels play a key role in many Ca^2+^-dependent signaling pathways, including those associated with the metastatic cascade such as EMT, migration, invasion and tumor vascularization (Iamshanova et al., 2017). In response to different environmental challenges during the metastatic cascade, like hypoxic, acidic and mechanical cues, cancer cells “re-program” TRPs expression and “misuse” their functions in order to put in place and sustain a more aggressive, metastatic phenotype. However, although most of the TRP-mediated pathways involved in cancer progression are due to alteration of Ca^2+^ homeostasis, it has been also demonstrated an involvement of these channels independent from their Ca^2+^ permeability. As an example, TRPM7 regulation on cell migration is mainly due to Mg^2+^ influx through the channel (Abed and Moreau, 2009; Su et al., 2011). Similarly, the Na^+^-selective TRPM4 channel has been implicated in cancer migration, although it is inherently Ca^2+^-impermeable (Vennekens and Nilius, 2007; Cáceres et al., 2015; Holzmann et al., 2015). On the other hand, TRPs’ involvement in the metastatic cascade may also be pore-independent, extending the interest in TRPs beyond the field of ion channels (Vrenken et al., 2015). Indeed, TRPM7 promotes many of its biological effects through its peculiar intrinsic kinase activity (Desai et al., 2012; Faouzi et al., 2017; Cai et al., 2018). Moreover, we recently demonstrated the role of TRPM8 in inhibiting vascular endothelial cell migration, which is independent from the pore function of the channel (Genova et al., 2017).

It is therefore evident that, despite the countless advances made in recent years in the study of TRP channels, there are still many aspects to be explored in order to better characterize the main TRP-mediated pathways involved in tumor progression and thus be able to develop new cancer therapies that use TRPs as therapeutic targets.

### Small GTPases

The family of small GTPases is composed of a large group of structurally and functionally related proteins, subgrouped into six families: Ras, Rho, Arf, Rab, Ran, and RGK. Among them, Ras-like and Rho-like GTPases are the most well characterized. Mechanistically, small GTPases are molecular switches that cycle between an active GTP- bound form and an inactive GDP-bound form. More specifically, these enzymes, once bound to GTP, can catalyse its hydrolysis to GDP and this reaction then results in a conformational change which causes the inactivation of the proteins (Vetter and Wittinghofer, 2001; Cherfils and Zeghouf, 2013). The cycling between GTP- and GDP-bound states is tightly regulated by specific GTPases activating protein (GAPs), which act as negative regulators, promoting the GDP-bound state by increasing the hydrolysis activity of small GTPases, and by guanine exchange factors (GEFs), which act as positive regulators, guiding the replacement of the hydrolysed GDP for a GTP, thus promoting the enzyme active state (Mishra and Lambright, 2016). Moreover, a third family of regulatory proteins called guanine-nucleotide dissociation inhibitors (GDIs) inhibits small GTPases activity by controlling their intracellular localization: GDIs bind to GTPases in their inactive GDP-bound state and sequester them in the cytosol, thus preventing their translocation to intracellular membranes, where activation occurs (Mishra and Lambright, 2016). Indeed, it has been shown that spatial and temporal distributions of the different small GTPases, as well as of their regulators, are important determinants in signaling by small GTPases, determining many aspects of cell behavior (Yarwood et al., 2006; Cherfils and Zeghouf, 2013; Moissoglu and Schwartz, 2014).

The conformational changes following the binding to GTP allow the association of small GTPases with a large number of potential effector proteins such as enzymes and scaffold proteins, which mediate the specific biological effects of each GTPases. Thanks to their ability to interact with a wide number of downstream targets and to co-ordinately activate several molecular processes required for a particular cellular response, small GTPases function as signaling switches in numerous cellular processes. In general, it has been found that Ras-like GTPases are mainly implicated in regulating cell cycle, differentiation and proliferation, (Shields et al., 2000), whereas Rho-like GTPases are mainly involved in cell morphology, cytoskeletal dynamics and cell polarity (Ridley, 2001). Moreover, Rab GTPases play a key role in many cellular functions, by controlling intracellular trafficking between organelles through vesiculotubular carriers and thus ensuring the spatiotemporal regulation of vesicle traffic (Stenmark, 2009). Considering the central role of Ca^2+^ signaling in many of these processes, it is not surprising that small GTPases activity was found to be strongly related to Ca^2+^ homeostasis (Aspenström, 2004). More specifically, their activation/inactivation may occur through Ca^2+^-dependent mechanisms acting on specific GEF/GAP proteins or directly on them. Moreover, some GTPases have revealed a direct influence on calcium signaling by regulating the activity of certain calcium channels, including TRPs, by itself or through their effectors (Koopman et al., 2003; Aspenström, 2004; Iftinca et al., 2007; Correll et al., 2008). Finally, several small GTPases collaborate with calcium signaling through the activation of specific Ca^2+^-related effectors involved in cellular processes, such as cell adhesion, cell migration and exocytosis (Cullen and Lockyer, 2002; Aspenström, 2004; Bader et al., 2004).

Small GTPases expression results dysregulated in several tumors and has been correlated with the progression of the disease (Sahai and Marshall, 2002; Svensmark and Brakebusch, 2019). Without a doubt, Ras is still the most studied small GTPase in cancer, due to the role played by mutated ras genes in the pathogenesis of human tumors (Bos, 1989; Hobbs et al., 2016; Li et al., 2018). Indeed, oncogenic mutations on H-ras, K-Ras, and N-Ras genes have been detected in several tumor types, although the incidence varies greatly (Bos, 1989; Prior et al., 2012; Li et al., 2018). Moreover, some members of the Ras superfamily like Rap1, resulted involved in cancer hallmarks such as migration and angiogenesis, due to their key role in integrin-mediated “inside-out” signaling events (Chrzanowska-wodnicka et al., 2008; Boettner and Van Aelst, 2009; Carmona et al., 2009). As regards the Rho superfamily, its role in many aspects of the metastatic cascade, including EMT, cell migration, invasion and angiogenesis, has been well established (Bryan and D’Amore, 2007; Ungefroren et al., 2018; Clayton and Ridley, 2020).

### TRPs-Small GTPases Interplay in Metastatic Cancer

#### Migration

One of the key steps in the metastatic cascade involves the acquisition of motility by cancer cells. This results from a complex coordination between cytoskeleton dynamics, cellular contractility and cell adhesion rearrangements. Cell migration is a dynamic process characterized by the cycling of four principal steps: after an initial phase in which cell spreading increases about twice with the generation of protrusions at the leading edge and cell adhesion to the ECM increases, then spreading is reduced and thanks to the generation of traction forces cell compacts and detaches at the trailing edge, allowing for cellular movement (Haws et al., 2016). Due to their role as key regulators of cytoskeletal dynamics and cell polarity, it is not surprising that Rho GTPases play a central role in controlling cell migration (Clayton and Ridley, 2020). This process is triggered by cell acquisition of a front-rear end polarity due to the formation of protrusive structures, called filopodia and lamellipodia, at the front edge, opposite to a retracting trailing edge (Llense and Etienne-Manneville, 2015). Filopodia consist of actin filaments organized as long parallel bundles and their formation is dependent on Rho GTPase Cdc42 activity, whereas lamellipodia result from the subsequent Rho GTPase Rac1-mediated branching of actin filaments. The extension of actin-based protrusions is accompanied by the formation at the leading edge of new adhesions that link integrins on the plasma membrane to the F-actin cytoskeleton through talin, vinculin, and focal adhesion kinases (FAKs) and which can mature into focal adhesions (FAs) in a process Rho GTPase RhoA- dependent. Contemporary, RhoA also mediates cytoskeletal rearrangements that lead to the formation of stress fibers, structures composed of bundles of actin and myosin II that have a high contractile capability. The force necessary to pull the cell body forward is engendered by the association of mature FAs with the end of stress fibers and by the actin-myosin cytoskeleton contraction. Finally, the disassembly of FAs in the rear of the migrating cells supports cell retraction at the trailing edge, allowing for cell detachment and movement.

**Figure 1:**
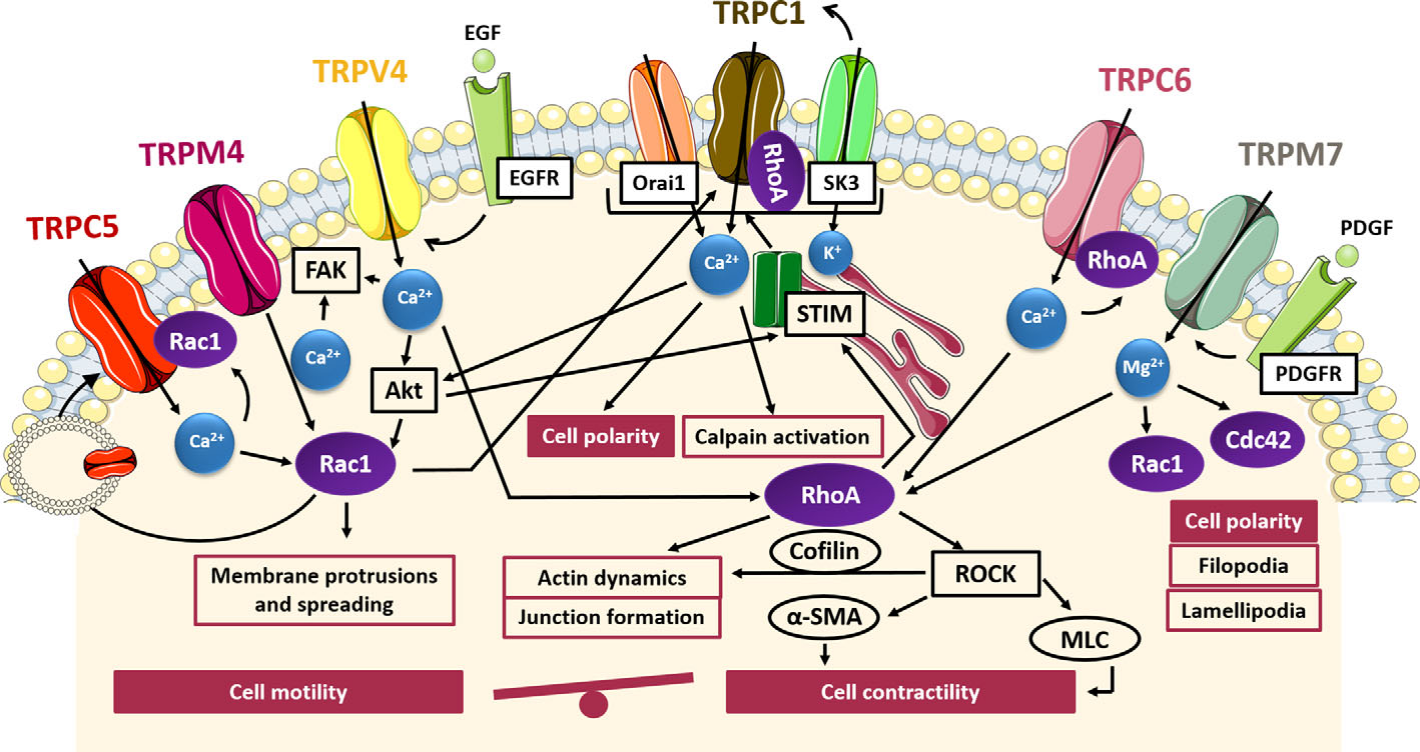
TRP- small GTPases signaling pathways interplay in cell migration. Cartoon depicting TRP channels signaling pathways affecting cell motility and contractility through GTPases. TRPC5, TRPM4 and TRPV4 induce the formation of protrusions and spreading *via* Rac1 activation in a Ca^2+^-dependent manner and at the same time Rac1 promote the translocation of TRPC5 into the plasma membrane; Rac1 and RhoA through SOCE activation induce TRPC1-mediated cell polarization for directional cell migration; TRPM7 control polarized cell movement through the regulation of Rac1 and Cdc42 in a Mg^2+^-dependent way; TRPM7, TRPV4, and TRPC6 contribute to actin dynamics and cell contractility through the Mg^2+^- or Ca^2+^-mediated activation of RhoA/ROCK pathways. FAK, focal adhesion kinase; Akt, protein-kinase B; EGF, epidermal growth factor; EGFR, epidermal growth factor receptor; PDGF, platelet-derived growth factor; PDGFR, platelet-derived growth factor receptor; Orai1, calcium release-activated calcium channel; SK3, small conductance calcium-activated potassium channel 3; STIM, stromal interaction molecule 1; ROCK, Rho-associated protein kinase; α-SMA, alfa-smooth muscle actin; MLC, myosin light chain.

A direct involvement of TRP channels activity in all processes associated with cell migration, including cytoskeletal rearrangement, FA turnover and cellular contractility, has been well established (Fiorio Pla and Gkika, 2013; Canales et al., 2019). Therefore, it is not surprising that several studies have highlighted a tight interplay between TRP channels and Rho GTPases in controlling cell migration during cancer progression. As depicted in **Figure 1**, this interplay is characterized by a bidirectional communication, in which TRP channels promote actin cytoskeleton reorganization through a cation-dependent activation of Rho GTPases, and, conversely, some small GTPases cause changes in TRP channel location, protein–protein interactions and channel gating, thereby modulating their function. More specifically, as described in the following paragraphs, TRPC5, TRPC6, TRPC1, TRPM7, TRPM4, and TRPV4 have been found to affect cell migration through a direct interaction with some small GTPases belonging to the Rho family.

Indeed, TRPC5 and TRPC6 channels have been identified as antagonist regulators of actin remodelling and cell motility in fibroblast and kidney podocytes, mediating the activation of Rac1 and RhoA, respectively (Tian et al., 2010). It has been shown that TRPC5 and TRPC6 trigger antagonistic and mutually inhibitory pathways associated with the maintenance of the balance between contractility and motility. In particular, TRPC5-mediated Ca^2+^ influx induces Rac1 activation, thereby enhancing motility and cell migration. Conversely, TRPC6-mediated Ca^2+^ influx stimulates an increase in RhoA activation, thereby promoting stress fibers formation, cell contractility and the subsequent inhibition of cell migration (Tian et al., 2010) or the disruption of podocytes architecture in glomerular renal diseases, which may lead to proteinuria (Jiang et al., 2011). Interestingly, the functional coupling between TRPC5 and TRPC6 with Rac1 and RhoA respectively, gives rise to two distinct molecular complexes predominantly localized to discrete membrane compartments, as detected by co-immunoprecipitation and immunofluorescence (Tian et al., 2010). Of note, the presence of constitutively active Rac1 has been shown to affect TRPC5 channel localization, leading to an increase in plasma membrane abundance of TRPC5 with respect to TRPC6, which, conversely, is predominant in cells expressing constitutively active RhoA. In this regard, RhoA seemed to have no effect on the dynamics of TRPC6 insertion into the membrane, whereas Rac1 was found to promote translocation of TRPC5 into the plasma membrane, according to other findings (Bezzerides et al., 2004). This close interdependence between TRPC5 and Rac1 could suggest a positive feedback mechanism in which the Rac1-mediated TRPC5 insertion from a vesicular pool into the cell membrane leads to enhanced TRPC5-mediated Ca^2+^ influx, which in turn triggers the activation of Rac1 and the subsequent migratory phenotype. Although a direct correlation between TRPC5/TRPC6 and Rac1/RhoA has not yet been established in cancer models, it is possible to speculate that a similar interplay may also occur in the regulation of cancer cells migration, taking into account the evidence for direct involvement of TRPC5 and TRPC6 in the increased migratory potential of several cell types (Greka et al., 2003; Xu et al., 2006; Rampino et al., 2007), including some tumors such as colon cancer and glioblastoma (Chigurupati et al., 2010; Chen L. et al. 2017; Chen Z. et al., 2017). However, whether these effects on tumor migration are dependent on the impact of TRPC5 and TRPC6 activity on cell contractility and motility remains to be clarified.

Similarly, TRPC1 has shown a correlation with an increased migratory phenotype in some tumors, such as glioblastoma, osteosarcoma, thyroid, pancreatic and colon carcinoma (Dong et al., 2010; Asghar et al., 2015; Huang et al., 2015; Guéguinou et al., 2016; Lepannetier et al., 2016). One of the key steps in cell migration is the establishment of a functional and morphological polarity along the axis of movement. In this context, it has been demonstrated that TRPC1 is localized to lipid raft domains at the leading edge of migrating cells and plays a role in determining their polarity and directionality (Fabian et al., 2008; Bomben et al., 2011; Huang et al., 2015). For instance, it has been shown that silencing of TRPC1 in transformed renal epithelial Madin–Darby Canine Kidney-Focus (MDCK-F) cells leads to a decrease in migration associated with a failure of cell polarization and an impaired lamellipodia formation. This effect is due to the partial loss of the local Ca^2+^ gradient at the front edge, needed to establish and maintain the axis of movement in migrating cells (Fabian et al., 2008). The same results were also shown in U2OS bone osteosarcoma cells, in which TRPC1 inhibition or knockdown correlates with a decrease in the percentage of polarized cells and the consequent reduction in cell migration (Huang et al., 2015). However, the link between TRPC1-mediated Ca^2+^ gradients and actin dynamics, as well as the possible involvement of TRPC1-mediated Rho GTPases activation in these processes has not been yet fully characterized. Nonetheless, a direct interaction between TRPC1 and RhoA has been characterized in intestinal epithelial cells (IECs) and in pulmonary arterial endothelial cells (Mehta et al., 2003; Chung et al., 2015). Indeed, TRPC1-RhoA interaction regulates TRPC1-mediated Ca^2+^ influx through SOCE, stimulating IECs migration after wounding (Chung et al., 2015). A reduction of RhoA/TRPC1 complexes, induced by downregulation or inactivation of either small GTPase or TRP channel, is associated with an inhibition of Ca^2+^ influx after store depletion and the decrease in wound healing after injury (Chung et al., 2015). Besides RhoA, a mechanism involving TRPC1 and Rac1 in promoting colon cancer cell migration has recently been proposed (Guéguinou et al., 2016). Indeed, in HCT-116 colon cancer cells TRPC1 and Rac1 are involved in a complex positive feedback loop in which EGF-induced SOCE activates both Rac1 and STIM1 through Akt pathway; in turn, STIM1 activation promotes translocation of TRPC1 and Orai1 into lipid rafts where SK3 is located and thereby triggers SOCE mediated by the complex SK3/TRPC1/Orai1. At the same time, Akt-mediated Rac1 activation enhances SOCE and thereby SOCE-dependent cell migration through Akt pathway, with subsequent lamellipodia formation and calpain activation. Taken together these data suggest a direct interplay between TRPC1 and Rho GTPases in controlling cell polarity and actin rearrangements during cancer cell migration.

TRPM7 is involved in directional migration in different cell types including migrating fibroblasts, osteoblasts, astrocytes and endothelial cells (ECs) (Abed and Moreau, 2009; Wei et al., 2009; Baldoli et al., 2013; Zeng et al., 2015). Similarly to TRPC1, TRPM7 has been shown to be involved in Ca^2+^ gradient formation, contributing to cell polarization and directional migration (Wei et al., 2009). In particular, TRPM7 is positively correlated with platelet-derived growth factor (PDGF)-induced Mg^2+^ influx as well as high-calcium microdomains “Ca^2+^ flickers”, most active at the leading lamella of migrating cells (Abed and Moreau, 2009; Wei et al., 2009). Interestingly, several data have shown that the effects of TRPM7 in controlling cytoskeleton and polarized cell movement are independent of its kinase activity and are associated with its channel function. This has been clearly demonstrated in fibroblasts and neuroblastoma cells where re-expression of TRPM7, as well as a kinase-inactive mutant of TRPM7 on knock out cells, reverted phenotypic changes in cell polarization enhancing cell spreading and adhesion (Clark et al., 2008; Su et al., 2011). In particular, it has been shown that Mg^2+^ plays a central role in TRPM7-mediated control of directional migration in fibroblasts and osteoblasts (Abed and Moreau, 2009; Su et al., 2011). The effects observed by TRPM7 depletion in cell morphology, disruption of actin filaments and myosin fibers and a decrease in the number of FAs, correlates with decreased activity of RhoA GTPase, suggesting a role for TRPM7 in RhoA regulation (Su et al., 2011). Besides its interaction with RhoA, TRPM7 is also functionally coupled with Rac1 and Cdc42: indeed, TRPM7 knockdown prevents Rac1 and Cdc42 activation, with a subsequent deficiency in their ability to form lamellipodia and impaired polarized cell movements (Su et al., 2011). Although Rho GTPases, differing from Ras and Rab proteins, do not require Mg^2+^ for high-affinity nucleotide binding, it has been shown that Mg^2+^ plays a role in regulating nucleotide binding and hydrolysis kinetics in the GEF- and GAP-catalyzed reactions of Rho family GTPases (Zhang et al., 2000). In particular, RhoGAPs exploit Mg^2+^ to achieve high catalytic efficiency and specificity and, conversely, RhoGEFs are negatively regulated by free Mg^2+^, since the presence of Mg^2+^ significantly decreases the intrinsic dissociation rates of the nucleotides. This finding may suggest that one role of GEFs is to displace bound Mg^2+^ from Rho proteins in order to efficiently perform their function and dissociate nucleotide from Rho GTPases (Zhang et al., 2000). Taken together this data revealed an interesting and so far little investigated interplay between TRP channel and Rho GTPases in controlling cell migration mediated by Mg^2+^ homeostasis.

More recently, another member of the TRPM subfamily, TRPM4, has been recognized as the first TRP channel to be part of the adhesome that is the set of protein components of FAs required for migration and contractility (Cáceres et al., 2015). Indeed, it has been shown that TRPM4 localises to FAs in different cell types and that its suppression impaires FAs relocation and lamellipodia formation, leading to a reduced cellular spreading and migration in mouse embryonic fibroblasts. Actually, FA turnover plays a key role in cell migration, contributing to the generation of the traction forces necessary for cellular movements. TRPM4-mediated effects on cell migration are at least partially due to the activation of Rac1 GTPase. Indeed, it has been observed that upon silencing TRPM4 the serum-induced activation of Rac1 and lamellipodial distribution are significantly reduced, suggesting a direct cooperation between TRPM4 and Rac1 in the regulation of cellular spreading. Moreover, it has been demonstrated that TRPM4 pharmacological inhibition caused retarded skin wound healing *in vivo*, affecting cell contractility (Cáceres et al., 2015). TRPM4 has been found to affect the migratory behaviour of many cell types, including prostate cancer cells (Holzmann et al., 2015). Although TRPM4 itself is Ca^2+^-impermeable, its contribution to cell migration through the regulation of intracellular Ca^2+^ signaling has been well established (Vennekens and Nilius, 2007). For instance, in androgen-insensitive prostate cancer cells, it has been shown that TRPM4 acts as an important negative feedback regulator of SOCE, thus promoting cell migration (Holzmann et al., 2015). However, further investigations are needed to deepen the knowledge of the molecular mechanism underlying the pro-migratory effect of TRPM4 on prostate cancer cell migration.

To date, several experimental pieces of evidence have revealed a critical role of TRPV4 in regulating the migratory properties of many tumors, including liver, breast and gastric cancer (Vriens et al., 2004; Lee et al., 2016; Xie et al., 2017). However, little is known about the molecular mechanism driving this process. Nonetheless, it has been shown that TRPV4 is involved in the dynamics of trailing adhesions, likely through an interplay with other cation channels or proteins present at the FA sites (Mrkonjić et al., 2015). A direct correlation between TRPV4 and RhoA/ROCK pathways has been revealed in cardiac fibroblast remodelling and myofibroblast contraction. In particular, it has been shown that after stimulation with growth factors, TRPV4 contributes to cell contractility by increasing the actin protein α-SMA expression and incorporation into stress fibers, through the Ca^2+^-mediated activation of RhoA/ROCK pathways (Tomasek et al., 2006; Adapala et al., 2013; Thodeti et al., 2013). Another study reported a role of TRPV4 in the modulation of adherent-junctions, mediated by the TRPV4-dependent activation of Rho GTPases, thereby promoting actin fibers organization and junctions formation (Sokabe and Tominaga, 2010). Furthermore, the exogenous up-regulation of TRPV4 in breast cancer has been shown to aid actin dynamics and lead to higher activation of cofilin, a downstream protein effector of RhoA/ROCK pathways that promotes actin filaments depolymerisation, thus conferring cellular “softness” and promoting transendothelial migration (Lee et al., 2016). Accordingly, TRPV4 knockdown reduced migration, invasion and transendothelial migration in breast cancer cells (Lee et al., 2016). Finally, a recent study of Ou-yang and co-worker (Ou-yang et al., 2018) has described the Akt/Rac1 signaling pathway through which TRPV4 promotes migration and invasion in glioma cancer cells (Ou-yang et al., 2018). Mechanistically, agonist-mediated TRPV4 activation promoted the activation of Rac1 by targeting Akt for phosphorylation, thus enhancing glioma cell migration and invasion (Ou-yang et al., 2018). Accordingly, in gastric cancer, TRPV4-mediated Ca^2+^ influx promotes cell migration through the activation of the downstream Akt/β-catenin pathways (Xie et al., 2017). Collectively, these data support a direct interplay between TRPV4 and small GTPases in controlling cytoskeletal remodeling aimed to confer migratory phenotypes.

#### Invasion

In order to reach lymph- and bloodstreams and to colonize sites distant from the primary tumor, cancer cells have to acquire, beyond migratory phenotype, the ability to degrade ECM. Consequently, invasion is another key step of the metastatic cascade. Invasiveness of cancer cells comes from their ability to produce special protrusions called invadopodia, which are actin-rich protrusions of the plasma membrane with proteolytic activity. Once matured, invadopodia recruit proteolytic enzymes such as membrane-matrix metalloproteinases (MMPs), which are endopeptidases able to locally degrade ECM, allowing cell invasion. Among them, MMP-2 and MMP-9 are considered the most important in metastasis, since they were often aberrantly expressed in tumors (Jabłońska-trypuć et al., 2016). Both TRP channels and small GTPases have been implicated in increased tumor invasiveness through the induction of MMPs expression (Betson and Braga, 2003; Yang and Kim, 2020). Indeed, TRPM2, TRPM7, TRPM8, TRPV2, and TRPC1 have shown to cause upregulation of MMP9 in a Ca^2+^-dependent manner in gastric, bladder, oral squamous, prostate and thyroid cancer cells respectively (Monet et al., 2010; Okamoto et al., 2012; Asghar et al., 2015; Cao et al., 2016; Chen L. et al. 2017; Chen Z. et al., 2017; Almasi et al., 2019). Additionally, TRPM2, TRPM7, and TRPC1 activity have been also correlated with MMP2 production in gastric, lung, pancreatic and thyroid cancer (Asghar et al., 2015; Rybarczyk et al., 2017; Liu et al., 2018; Almasi et al., 2019). On the other hand, Rho and Rac GTPases activation has been correlated with increased MMPs expression in different cancer cell types (Zhuge and Xu, 2001; Abécassis et al., 2003; Santibáñez et al., 2010; Jacob et al., 2013).

**Figure 2:**
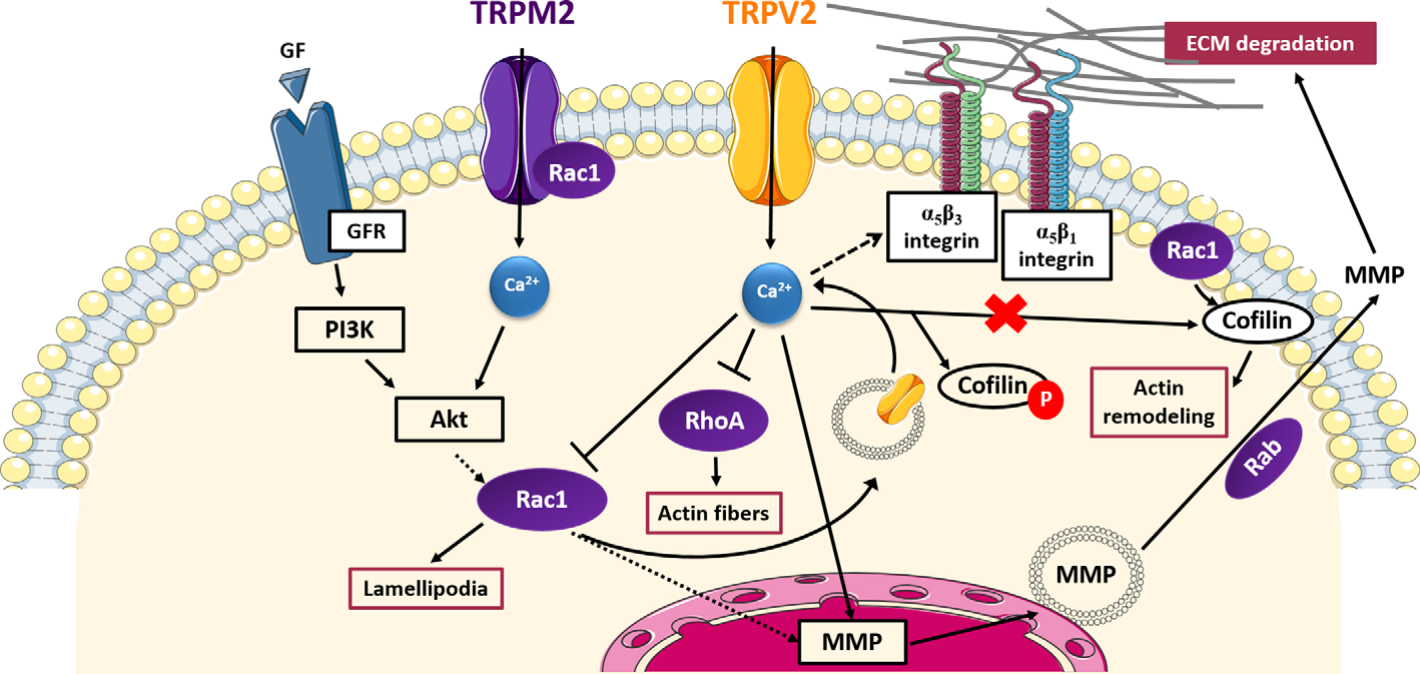
TRP- small GTPases signaling pathways interplay in cell invasion. Cartoon depicting TRP channels signaling pathways affecting cell invasiveness through GTPases. Rac1 promotes the translocation of TRPV2 into the plasma membrane and thus TRPV2-mediated increased in MMPs expression; TRPV2 affects also cell adhesion and invasion interfering with integrin-mediated signalling and inhibiting Rac1, RhoA, and cofilin activation by Rac1 in a Ca^2+^-dependent manner; TRPM2 and Rac1 physically interact with each other, mutually influencing their activity and lead to an increase in MMPs production; MMPs exocytosis is mediated by the Rab superfamily of small GTPases. GF, growth factor; GFR, growth factor receptor; PI3K, phosphoinositide-3 kinase; Akt, protein-kinase B; MMP, membrane-matrix metalloproteinase; ECM, extracellular matrix.

TRPV2 and TRPM2 have been shown to affect cell invasiveness through signaling pathways involving small GTPases, which are summarized in **Figure 2**.

In particular, TRPV2 has been found to positively correlate with prostate cancer (PCa) invasiveness, promoting PCa progression to the aggressive castration-resistant stage (Monet et al., 2010). More specifically, it has been shown that siRNA-mediated silencing of TRPV2 leads to a decrease in MMP-2 and MMP-9 expression, reducing growth and invasive properties of PC3 prostate tumors established in nude mice xenografts (Monet et al., 2010). The mechanism by which the Ca^2+^ influx mediated by TRPV2 is linked with the up-regulation of MMPs has not been characterized. However, another study has revealed that TRPV2 trafficking to the plasma membrane correlates with enhanced cell migration and invasion in PCa, *via* the phosphoinositide 3-kinase (PI3K) pathway (Oulidi et al., 2013). Taking into account evidence of a PI3K-mediated activation of Rac1 in several tumor models (Haws et al., 2016; Ungefroren et al., 2018) and considering that Rac1 has been found to regulate TRPV2 intracellular trafficking in fibrosarcoma cells (Nagasawa and Kojima, 2015), it is possible to speculate that in PCa a PI3K-mediated Rac1 activation may allow the translocation to the plasma membrane of the “*de novo*” expressed TRPV2 in PC3 cells, thus giving rise of the TRPV2-mediated increase of cytosolic Ca^2+^ concentration responsible for MMPs overexpression. Confirming this hypothesis, Rac1 has been related to the up-regulation of MMP-2 and MMP-9 in fibrosarcoma (Zhuge and Xu, 2001) and transformed keratinocytes (Santibáñez et al., 2010), respectively. Unlike in PCa, TRPV2 has been found to suppress the invasiveness of fibroblast-like synoviocytes (FLS), which have an aggressive and invasive behaviour resembling that of cancer cells (Laragione et al., 2015). TRPV2 activation has been associated both *in vitro* and *in vivo* with reduced cell invasiveness and a down-regulation of the IL-1β-induced expression of MMP-2 and MMP-3 (Laragione et al., 2015). More recently, the cell signaling events mediating this TRPV2 suppressive activity have been characterized (Laragione et al., 2019). Interestingly, a direct interplay between TRPV2 channel function and RhoA/Rac1 GTPases activity in suppressing FLS cell invasion has been proposed (Laragione et al., 2019). In particular, it has been shown that, upon stimulation with the commercially-available TRPV2-specific agonist O1821, the channel nearly disappears from the plasma membrane, as well as integrins αν, β1, and β3, involved in cell binding to ECM. Concomitantly, a decrease in cell adhesion and the number of thick actin filaments and a reduction in lamellipodia formation are observed. Mechanistically, it has been found that O1821-induced TRPV2 activation causes a decrease in both RhoA and Rac1 activation, giving rise to the observed inhibition of actin filaments and lamellipodia formation, respectively. Moreover, TRPV2 activation significantly increases levels of phosphorylated (inactive) cofilin and affects the localization of active cofilin keeping it in the cytosol away from cell protrusions and lamellipodia, in which normally it exerts its function on actin remodeling, upon activation by Rac1. Considering the active involvement of Rac1 in TRPV2 trafficking found by Nagasawa and Kojima (2015), Rac1 inhibition through TRPV2 described by Laragione *et al*. might also explain the observed reduction of TRPV2 expression on the plasma membrane after channel stimulation, suggesting an intriguing negative feedback loop between TRPV2 and Rac1 regulation. Indeed, TRPV2 activation on the plasma membrane may inhibit in a Ca^2+^-dependent manner Rac1 activation, which in turn resulted in a decrease of TRPV2 expression on the cell surface. Taken together these data account for a possible two-sided interplay between Rac1 and TRPV2, based on which TRPV2 may exert both pro- and anti-invasiveness activities depending on cell type. In the first case Rac1 is activated by the PI3K pathway, thus allowing the overexpression of the constitutively active TRPV2 on the plasma membrane and the increased calcium flow responsible for MMPs overexpression and the increased cell invasiveness (Nagasawa and Kojima, 2015); in the second case TRPV2, upon activation by external stimuli, causes Rac1 inactivation and the inhibition of pro-invasive intracellular pathways as well as the expression of TRPV2 in the membrane through a negative feedback mechanism (Laragione et al., 2019). Therefore, in an interesting interchangeable relationship, TRPV2 may act as either modulator or effector of Rac1, depending on cell type, thus reflecting the bivalent activity showed by the channel in cell invasiveness.

TRPM2 offers another potential field of investigation on TRP-small GTPase relationship in cancer cells’ ability to invade surrounding tissues. In fact, it has been recently proven that TRPM2 causes an increase in MMP-9 production in gastric cancer (Almasi et al., 2019). As in the case of TRPV4, the function of TRPM2 appears to be regulated by the Akt pathway, known to regulate the activity of several GTPases including Rac1 (Ho et al., 2010), whose interaction with TRPM2 has been already well established in response to oxidative stress (Gao et al., 2014) (**Figure 2**). In this regard, it has been demonstrated that TRPM2 and Rac1 physically interact with each other, mutually influencing their activity, similarly to what was observed between TRPC5 and Rac1 in podocytes previously described (Bezzerides et al., 2004; Tian et al., 2010). Thus, we could speculate a TRPM2-Akt-Rac1 axis in the modulation of MMP-9 expression in gastric cancer.

#### Tumor Vascularization

**Figure 3:**
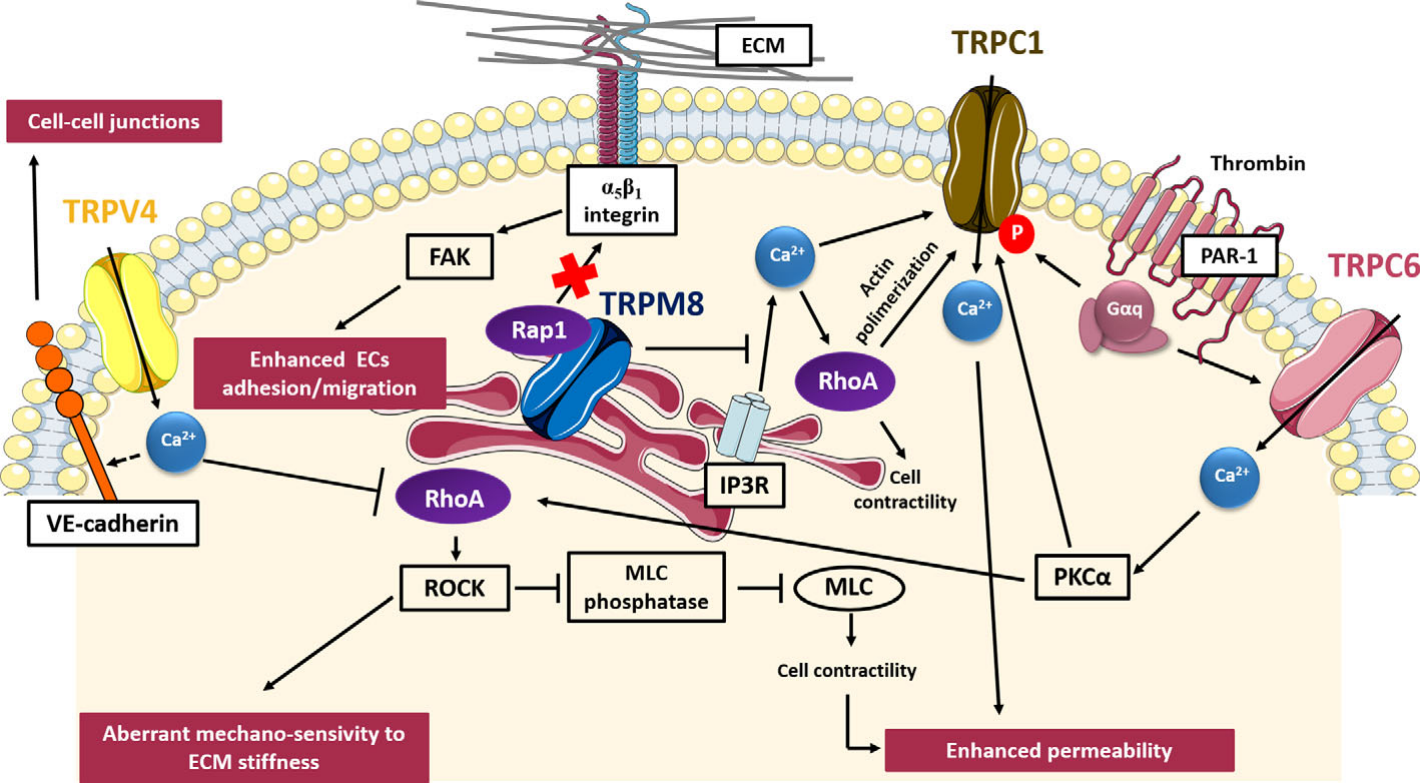
TRP- small GTPases signaling pathways interplay in aberrant tumor vascularization. Cartoon depicting TRP channels signaling pathways affecting tumor vascularization through GTPases. TRPV4 induces aberrant mechano-sensivity to ECM through the Ca^2+^-dependent inhibition of Rho/ROCK pathway; TRPC1 and TRPC6 enhance vessels permeability affecting cell contractility through a Ca^2+^-mediated regulation of RhoA; on the contrary, TRPM8 exerts a protective role in tumor vasculature permeability, inhibiting the store-operated RhoA activation and subsequent cell contraction; TRPM8 also inhibits ECs adhesion and migration, impairing activation of β_1_-integrin through the intracellular retention of Rap1. VE-cadherin, vascular E-cadherin; FAK, focal adhesion kinase; ROCK, Rho-associated protein kinase; MLC, myosin light chain; PKCα, protein kinase C alpha; IP3R, inositol trisphosphate receptor; PAR-1, protease-activated receptor-1.

Tumor vascularization, is a critical step in the metastatic cascade since the formation of new blood vessels is crucial not only to provide sufficient oxygen and nutrients and thus promoting the continuous growth of tumors but also to drive tumor spread and metastasis. Tumor vascularization, promoted by the tumor cells themselves through the secretion of several growth factors, results aberrant and leads to the formation of new vessels characterized by abnormal morphology, irregular blood flow, and distribution, non-uniform pericyte coverage and hyper-permeability (Carmeliet and Jain, 2000; Carmeliet and Jain, 2011). TRP channels are widely expressed within vascular ECs and several data correlate aberrant TRP channels expression and/or activity with tumor vascularization, thanks to their high sensitivity to both pro-angiogenic signals and subtle changes in the local microenvironment (Brossa et al., 2019; Negri et al., 2020; Scarpellino et al., 2020). TRP channels have been related to critical steps of tumor vascularization, including cell adhesion, cell migration, enhanced permeability and *in vitro* angiogenesis (Fiorio Pla and Gkika, 2013). Similarly, several Rho and Ras small GTPases, thanks to their contribution to actin dynamics, cell contractility and integrin-mediated “inside-out” signaling events, have been found to be dysregulated during tumor vascularization (Carmona et al., 2009; Chrzanowska-Wodnicka, 2010). Some common pathways involving both TRP channels and small GTPases in determining aberrant tumor vascularization have been described, such as those concerning TRPV4, TRPM8, TRPC1, and TRPC6, depicted in **Figure 3**.

TRPV4 has been the first TRP channel to be clearly implicated in tumor angiogenesis, although it can have both pro-angiogenic and anti-angiogenic effects depending on tumor type. In particular, TRPV4-mediated Ca^2+^ signals are implicated in tumor-derived endothelial cells (TECs) migration *via* a membrane-stretch activated arachidonic acid release and subsequent actin remodeling and TRPV4 insertion in the plasma membrane (Fiorio Pla et al., 2012). These data are in accordance with previous results showing that TRPV4 is required for shear stress EC reorientation in an integrin-dependent manner (Thodeti et al., 2009). However, in different TECs models, TRPV4 results to be down-regulated as compared to normal tissues. Moreover, this down-regulation is correlated with aberrant mechano-sensitivity of TECs towards ECM stiffness and subsequently with enhanced cell motility and abnormal angiogenesis. Subcutaneous injection of Lewis Lung Carcinoma cells in TRPV4 knockout mice leads to increased vascular density, higher vessel diameter, and reduced pericyte coverage, overall resulting in enhanced tumor growth (Adapala et al., 2016). More recently, it has also been shown by the same group that TRPV4 silencing causes a significant decrease in VE-cadherin expression at cell-to-cell junctions, with the subsequent increase in vascular leakage (Cappelli et al., 2019). Accordingly, overexpression or pharmacological stimulation of TRPV4 with GSK1016790A has been shown to lead to a “normalization” of the vascular endothelium, enhancing the permeability of chemotherapeutic drugs, and basically blocking tumor growth (Adapala et al., 2016). In particular, TRPV4-mediated “normalization” of aberrant capillary-like tubules *in vitro* is achieved by restoring the mechano-sensitivity of ECs toward ECM stiffness through the blockade of basal Rho activity (Adapala et al., 2016). In this contest, it was previously shown that the aberrant TECs mechano-sensitivity of TECs in response to ECM stiffness and cyclic strain results, at least partially, due to constitutively high level of Rho/ROCK basal activities (Ghosh et al., 2008). Moreover, ECs isolated from TRPV4 knockout (KO) mice display higher basal Rho activity as compared to EC WT and the inhibition of the Rho/ROCK pathway in TRPV4KO mice results in vessel normalization, confirming the role of TRPV4 as an important modulator of Rho activity also *in vivo* (Thoppil et al., 2016).

In agreement with other recent findings which have highlighted non-conducting functions of TRP channels in many processes, including the regulation of cytoskeletal dynamics (Vrenken et al., 2015), our recent study (Genova et al., 2017) has unveiled that TRPM8 inhibits ECs migration in a Ca^2+^-independent manner. TRPM8 is mainly localized in the endoplasmatic reticulum (ER) in ECs and its expression results remarkably down-regulated in breast TECs (BTECs) as compared to healthy human microvascular ECs (HMECs) and human umbilical vein ECs (HUVECs) (Genova et al., 2017). Mechanistically, TRPM8 inhibits ECs migration and spheroid sprouting by trapping Rap1 intracellularly, thereby preventing its relocation toward the plasma membrane, which is required to activate β_1_-integrin signaling. Inactive Rap1 (GDP- bound) physically interacts with the N-terminal tail of TRPM8 and interestingly, its retention within the cytosol occurs also in the presence of a TRPM8 pore mutant, demonstrating that TRPM8 inhibitory effects on ECs migration and adhesion by trapping Rap1 are independent from its channel function (Genova et al., 2017). Curiously, TRPM8 activation through specific channel agonists (icilin and menthol) or endogenous activators such as prostate-specific antigen (PSA), significantly reduces the amount of active Rap1-GTP bound and enhanced its inhibitory effect on migration, thus raising questions about the possible effects of agonists on TRPM8 besides pore gating. TRPM8-mediated inhibition of Rap1 cytoplasm–plasma membrane trafficking impairs the activation of inside-out signaling pathway known to trigger the conformational activation of β_1_-integrin and, consequently, cell adhesion and migration. Indeed, icilin-stimulated TRPM8 significantly inhibits active-β_1_ integrins as revealed by immunofluorescence assays. In addition, agonist-induced TRPM8 stimulation leads to a significant decrease in FAK phosphorylation, suggesting the involvement of FAK as a downstream effector of the β_1_-integrin pathway affected by TRPM8 (Genova et al., 2017). This study has highlighted how TRP channels may regulate GTPases activity not only by the generation of local Ca^2+^ fluxes, but also acting through physical interactions which affect their intracellular localization and thus their activity. Indeed, as well as TRP channels, also small GTPases are used for spatial and temporal control of cell behavior, as reported by several studies (Yarwood et al., 2006; Wang et al., 2012; Moissoglu and Schwartz, 2014). Another evidence of a TRPM8 interplay with small GTPases in controlling ECs behavior comes from a study of Sun and coworkers (Sun et al., 2014), which has revealed a role of TRPM8 in vasoconstriction and hypertension through attenuating RhoA/Rho kinase pathway. In particular, the authors have shown that TRPM8 activation by menthol attenuates vasoconstriction by inhibiting the RhoA/ROCK pathway in wild-type mice, but not in TRPM8KO mice (Sun et al., 2014). TRPM8 effect is associated with inhibition of intracellular calcium release from the sarcoplasmic reticulum, thus reducing Ca^2+^-mediated activation of RhoA/ROCK and ECs contraction. Since several experiments have correlated ECs contraction with vessel permeability (Hicks et al., 2010), this study could suggest a protective role of TRPM8 not only in ECs migration, but also in tumor vasculature permeability.

The increased permeability shown by tumor vessels may be induced by several factors, including hypoxia and inflammatory signals, such as thrombin. In this regard, TRPC6 and TRPC1 have been implicated in thrombin-induced hyper-permeabilization of ECs through the RhoA/ROCK signaling pathway. Thrombin is a serine-protease which binds specifically to the protease-activated receptor-1 (PAR-1) on endothelial cell surface, inducing a signaling cascade, which results in an increase in ECs contraction, changes in cell shape and finally, in the development of tiny inter-endothelial junctional gaps that lead to increased endothelial permeability. Interestingly, TRPC6 activation is promoted by thrombin stimulation through the α subunit of G protein-coupled receptors (Gα_q_) (Singh et al., 2007). Then, TRPC6-mediated Ca^2+^ influx leads to the activation of PKCα and thereby induces RhoA activity and ECs contraction, with subsequent cell shape changes, inter-endothelial gaps formation and increase in EC permeability (Singh et al., 2007). Indeed, upon thrombin stimulation it has been shown that RhoA activates its downstream effector ROCK, which in turn promotes myosin light chain (MLC) phosphatase regulatory subunit phosphorylation, reducing its phosphatase activity (Birukova et al., 2004). TRPC6 have also been found to play a central role in determining the angiogenic potential of glioma cells, since its inhibition affected EC tube formation *in vitro* by reducing the number of branch points (Chigurupati et al., 2010). Moreover, TRPC6 has been shown to exert pro-angiogenic effects by affecting vascular endothelial growth factor (VEGF)-induced calcium influx in ECs. Although there are not robust data on GTPases-mediated TRPC6 functions in angiogenesis, recently Zahra and coworkers defined an important role of RhoA in ECs proliferation, migration, invasion and sprouting triggered by important angiogenesis inducers, including VEGF (Zahra et al., 2019), thus suggesting a possible interplay between TRPC6 and RhoA in VEGF-induced angiogenesis.

Similarly to TRPC6, also TRPC1 has been found to have a role in increasing ECs permeability through RhoA activation in response to thrombin stimulation. In this regard, an intriguing mechanism depicting RhoA as a TRPC1 modulator in human pulmonary arterial endothelial cells has been proposed by Mehta and co-worker (Mehta et al., 2003). They showed that RhoA, upon thrombin-induced activation, forms a complex with IP_3_R and TRPC1, which then translocates to the plasma membrane, where TRPC1 can mediate a store depletion-induced Ca^2+^ entry and the resultant increase in endothelial permeability. Moreover, it has been demonstrated that RhoA-induced TRPC1-IP_3_R association is dependent on actin filament polymerization, since its inhibition hinders both the association and Ca^2+^ entry (Mehta et al., 2003). Moreover, TRPC1 serine/threonine phosphorylation by PKCα is crucial for inducing Ca^2+^ influx and consequently EC permeability (Ahmmed et al., 2004). However, the mechanism by with PKCα integrates with RhoA in ECs to trigger TRPC1-mediated Ca^2+^ influx remained to be characterized. Nonetheless, considering the previously described role of PKCα as a downstream effector of TRPC6 (Singh et al., 2007), it is possible that the PKCα- dependent RhoA-induced TRPC1 activation gives rise to a positive feedback loop that overall leads to a persistent increase endothelial permeability. This study provides an example of pathway in which small GTPases do not act as TRP Ca^2+^-dependent effectors, but rather as modulators of TRP channel activity, influencing protein-protein interactions and channel gating, and thus corroborating the bidirectional nature of this complex interplay.

### Conclusions and Perspectives

TRPs and small GTPases show a direct interplay in cancer progression, characterized by a bidirectional communication, whereby TRP channels have been shown to affect small GTPases activity *via* both Ca^2+^-dependent or Ca^2+^–independent pathways, and conversely some small GTPases may affect TRP channels activity through the regulation of their intracellular trafficking to the plasma membrane or acting directly on channel gating (**Table 2**). In most cases, TRP-GTPase interaction is mediated by Ca^2+^ signals, triggered by TRP-mediated Ca^2+^ influx through the plasma membrane induced by growth factors, specific ligands or mechanical stimuli. In some specific cases, such as TRPM7, TRP-mediated small GTPases activation/inhibition may be supported by Mg^2+^ rather than Ca^2+^ homeostasis regulation. Moreover, also Ca^2+^-impermeable TRP channels like TRPM4 have been found to regulate small GTPases activity, probably through an indirect control on other Ca^2+^-signaling pathways. Finally, alternative regulatory pathways that go beyond the canonical ones involving cation homeostasis have been characterized for TRP-mediated small GTPases regulation. For instance, it has been shown that some TRPs, such as TRPM8 in ECs, may act similarly to a GDI-like protein, inhibiting small GTPases activity by physically trapping and restraining them within a specific cellular compartment and thus preventing their switch to the active form that generally occurs at the plasma membrane. Although in most cases small GTPases act as TRP effectors, there are some evidence about the role of small GTPases as modulators of TRP channel activity. Indeed, it has been shown that some small GTPases may affect TRPs channel activity by influencing channel trafficking, gene expression, protein-protein interactions and channel gating. In some cases, positive feedback loop mechanisms, wherein TRP channels activate small GTPases which in turn increase TRPs insertion to the plasma membrane, have also been described. In addition, some studies have highlighted that effector/modulator roles may be interchangeable between TRPs and small GTPases depending on cell type. For instance, it has been shown that Rac1 may act as either modulator or effector of TRPV2 activity, thus determining opposite effects on cell invasion, depending on cell type.

**Table 2:** TRPs-small GTPases relationship in metastatic cancer hallmarks.

Hallmark	TRP Channel		GTPase	Biological Effect	Reference
Migration	TRPC1	**Ca^2+^** **←**	RhoA	↑	(Mehta et al., 2003; Chung et al., 2015)
		**Ca^2+^** **↔**	Rac1	↑	(Guéguinou et al., 2016)
	TRPC5	**Ca^2+^** **↔**	Rac1	↑	(Bezzerides et al., 2004; Tian et al., 2010)
	TRPC6	**Ca^2+^** **→**	RhoA	↓	(Tian et al., 2010)
	TRPM4		Rac1	↑	(Cáceres et al., 2015)
	TRPM7	**Mg^2+^** **→**	RhoA	↑	(Su et al., 2011)
		**Mg^2+^** **→**	Rac1 Cdc42		(Su et al., 2011)
	TRPV4	**Ca^2+^** **→**	RhoA	↑	(Tomasek et al., 2006; Adapala et al., 2013; Thodeti et al., 2013; Lee et al., 2016)
		**Ca^2+^** **→**	Rac1	↑	(Ou-yang et al., 2018)
Invasion	TRPM2	**Ca^2+^** **↔**	Rac1	↑	(Gao et al., 2014)
	TRPV2	**←**	Rac1	↑	(Nagasawa and Kojima, 2015)
		**Ca^2+^** **┤**	RhoA/ Rac1	↓	(Laragione et al., 2019)
Aberrant tumor vascularization	TRPC1	**←**	RhoA	↑	(Mehta et al., 2003)
	TRPC6	**Ca^2+^** **→**	RhoA	↑	(Singh et al., 2007)
	TRPM8	**┤**	Rap1	↓	(Genova et al., 2017)
		**┤**	RhoA	↓	(Sun et al., 2014)
	TRPV4	**┤**	Rho	↓	(Thoppil et al., 2016; Adapala et al., 2016)

**→**: TRP enhances GTPase activity; **←**: GTPase enhance TRP activity; **↔**: bidirectional activation; **┤**: TRP inhibits GTPase activity; ↑: increase; ↓: decrease.

In addition to the examples of a direct TRP-small GTPases interaction in metastatic cancer hallmarks reported in this review, many studies suggest other possible signal transduction pathways associated with tumor progression involving both TRPs and small GTPases. For instance, they have both been implicated in the regulation of MMPs production through pathways like IP3K/Akt and Hsp90α-uPA-MMP2 that offer many points of contact in between them (Koike et al., 2000; Zhuge and Xu, 2001; Yang et al., 2015; Luo et al., 2016; Rybarczyk et al., 2017; Liu et al., 2018). However, to date, a direct correlation between TRPs and small GTPases in these signal transduction pathways has not yet been established but remains to be deeper clarified. Moreover, several studies have reported a role of both these two superfamilies of molecules in promoting another key step of the metastatic cascade that is EMT. In response to the same growth factor/cytokines-induced signaling pathways both TRPs and small GTPases are able to induce the up-regulation of mesenchymal-like markers such as vimentin and the down-regulation of epithelial-like markers such as E-cadherin through the direct regulation of transcriptional factors including STAT3, Snail and Twist (Simon et al., 2000; Davis et al., 2014; Liu et al., 2014; Yang et al., 2015; Chen L. et al., 2017; Kim et al., 2018 Liu et al., 2019; Seiz et al., 2020). However, a synergistic cooperation between TRPs and Rho GTPases during the early stage of growth-factor induced EMT is still only speculation, since to the best knowledge of the authors a direct correlation between TRPs and small GTPases effects on EMT has not yet been characterized. In conclusion, the interplay between TRP channels and small GTPase in cancer progression is only partially investigated to date and further investigations are required to shed light on many other aspects of this interesting crosstalk in cancer not well known.

## Author Contributions

DG conceived the study. GC provided wrote and edited the manuscript and designed the figures. AP revised the manuscript. All authors contributed to the article and approved the submitted version.

## Funding

GC was supported by University of Torino as part of the PhD Program in Complex Systems for Life Sciences. DG is supported by the Institut National du Cancer (INCa- PLBIO14-213) and the Institut Universitaire de France (IUF). GC, AP, and DG were supported by the International Associated Laboratory (LAI) agreement (CaPancInv). AP was supported by grants from the Italian Ministry of Instruction, University and Research (MIUR), PRIN grant “Leveraging basic knowledge of ion channel network in cancer for innovative therapeutic strategies (LIONESS)” (grant number 20174TB8KW).

## Conflict of Interest

The authors declare that the research was conducted in the absence of any commercial or financial relationships that could be construed as a potential conflict of interest.

## Abbreviations

EC, endothelial cell; ECM, extracellular matrix; EGF, epidermal growth factor; EMT, epithelial to mesenchymal transition; ER, endoplasmatic reticulum; FA, focal adhesion; FAK, focal adhesion kinase; GAP, GTPases activating protein; GEF, guanine exchange factor; GDI, guanine-nucleotide dissociation inhibitors; GDP, guanosine diphosphate; GTP, guanosine triphosphate; GTPase, guanosine triphosphatases; MLC, myosin light chain; MMP, membrane-matrix metalloproteinases; PCa, prostate cancer; PI3K, phosphoinositide 3-kinase; PFGF, platelet-derived growth factor; TEC, tumor endothelial cell; TRP, transient receptor potential; VEGF, vascular endothelial growth factor.

## References

[B1] Abécassis I. Olofsson B. Schmid M. (2003). RhoA Induces MMP-9 Expression at CD44 Lamellipodial Focal Complexes and Promotes HMEC-1 Cell Invasion. Exp. Cell Res. 291 (2), 363–376. 10.1016/j.yexcr.2003.08.006 14644158

[B2] Abed E. Moreau R. (2009). Importance of Melastatin-like Transient Receptor Potential 7 and Magnesium in the Stimulation of Osteoblast Proliferation and Migration by Platelet-Derived Growth Factor. Am. J. Physiol. - Cell Physiol. 297 (2), 360–368. 10.1152/ajpcell.00614.2008 19474290

[B3] Adapala R. K. Thoppil R. J. Luther D. J. Paruchuri S. Meszaros J. G. Chilian W. M. (2013). Cellular Cardiology TRPV4 Channels Mediate Cardiac Fi Broblast Differentiation by Integrating Mechanical and Soluble Signals. J. Mol. Cell. Cardiol. 54, 45–52. 10.1016/j.yjmcc.2012.10.016 23142541 PMC3935769

[B4] Adapala R. K. Thoppil R. J. Ghosh K. Cappelli H. Dudley A. C. Paruchuri S. (2016). Activation of Mechanosensitive Ion Channel TRPV4 Normalizes Tumor Vasculature and Improves Cancer Therapy. J. Autism Dev. Disord. 35 (3), 314–322. 10.1097/CCM.0b013e31823da96d.Hydrogen PMC4948740 25867067

[B5] Ahmmed G. U. Mehta D. Vogel S. Holinstat M. Paria B. C. Tiruppathi C. (2004). Protein Kinase Cα Phosphorylates the TRPC1 Channel and Regulates Store-Operated Ca 2 Entry in Endothelial Cells *. J. Biol. Chem. 279 (20), 20941–20949. 10.1074/jbc.M313975200 15016832

[B6] Almasi S. Sterea A. M. Fernando W. Clements D. R. Marcato P. Hoskin D. W. (2019). TRPM2 Ion Channel Promotes Gastric Cancer Migration, Invasion and Tumor Growth through the AKT Signaling Pathway. Sci. Rep. 9 (1), 4182. 10.1038/s41598-019-40330-1 30862883 PMC6414629

[B7] Asghar M. Y. Magnusson M. Kemppainen K. Sukumaran P. Löf C. Pulli I. (2015). Transient Receptor Potential Canonical 1 (TRPC1) Channels as Regulators of Sphingolipid and VEGF Receptor Expression: Implications for Thyroid Cancer Cell Migration and Proliferation. J. Biol. Chem. 290 (26), 16116–16131. 10.1074/jbc.M115.643668 25971967 PMC4481213

[B8] Ashida S. Nakagawa H. Katagiri T. Furihata M. Iiizumi M. Anazawa Y. (2004). Molecular Features of the Transition from Prostatic Intraepithelial Neoplasia (PIN) to Prostate Cancer: Genome-Wide Gene-Expression Profiles of Prostate Cancers and PINs. Cancer Res. 64 (17), 5963–5972. 10.1158/0008-5472.CAN-04-0020 15342375

[B9] Aspenström P. (2004). Integration of Signalling Pathways Regulated by Small GTPases and Calcium. Biochim. Biophys. Acta - Mol. Cell Res. 1742 (1–3), 51–58. 10.1016/j.bbamcr.2004.09.029 15590055

[B10] Azimi I. Milevskiy M. J. G. Kaemmerer E. Turner D. Yapa K. T. D. S. Brown M. A. (2017). TRPC1 Is a Differential Regulator of Hypoxia-Mediated Events and Akt Signalling in PTEN-Deficient Breast Cancer Cells. J. Cell Sci. 130 (14), 2292–2305. 10.1242/jcs.196659 28559303

[B11] Bader M. F. Doussau F. Chasserot-Golaz S. Vitale N. Gasman S (2004). Coupling Actin and Membrane Dynamics during Calcium-Regulated Exocytosis: A Role for Rho and ARF GTPases. Biochim. Biophys. Acta - Mol. Cell Res. 1742 (1–3), 37–49. 10.1016/j.bbamcr.2004.09.028 15590054

[B12] Baldoli E. Castiglioni S. Maier J. A.M. (2013). Regulation and Function of TRPM7 in Human Endothelial Cells: TRPM7 as a Potential Novel Regulator of Endothelial Function. PloS One 8 (3), e598915. 10.1371/journal.pone.0059891 PMC3606311 23533657

[B13] Bernardini M. Pla A. F. Prevarskaya N. Gkika D. (2015). Human Transient Receptor Potential (TRP) Channels Expression Profiling in Carcinogenesis. Int. J. Dev. Biol. 59 (7–9), 399–406. 10.1387/ijdb.150232dg 26679952

[B14] Berridge M. J. Bootman M. D. Roderick H. L. (2003). CALCIUM SIGNALLING : DYNAMICS, HOMEOSTASIS AND REMODELLING. Nat. Rev. Mol. Cell Biol. 4 (7), 517–529. 10.1038/nrm1155 12838335

[B15] Betson M. Braga V. M.M. (2003). Tumor Progression : Small GTPases and Loss of Cell – Cell Adhesion. Bioessays 25 (5), 452–635. 10.1002/bies.10262 12717816

[B16] Bezzerides V. J. Ramsey I. S. Kotecha S. Greka A. Clapham D. E. (2004). Rapid Vesicular Translocation and Insertion of TRP Channels. Nat. Cell Biol. 6 (8), 709–720. 10.1038/ncb1150 15258588

[B17] Bidaux G. Roudbaraki M. Merle C. Crépin A. Delcourt P. Slomianny C. (2005). Evidence for Specific TRPM8 Expression in Human Prostate Secretory Epithelial Cells: Functional Androgen Receptor Requirement. Endoc.-Rel. Cancer 12 (2), 367–382. 10.1677/erc.1.00969 15947109

[B18] Birukova A. A. Smurova K. Birukov K. G. Kaibuchi K. Garcia J. G.N. Verin A. D. (2004). Role of Rho GTPases in Thrombin-Induced Lung Vascular Endothelial Cells Barrier Dysfunction. Microvascular 67, 64–77. 10.1016/j.mvr.2003.09.007 14709404

[B19] Boettner B. Van Aelst L. (2009). Control of Cell Adhesion Dynamics by Rap1 Signaling. Curr. Opin. Cell Biol. 21 (5), 684–693. 10.1016/j.ceb.2009.06.004 19615876 PMC2841981

[B20] Bomben V. C. Turner K. L. Barclay T. C. Sontheimer H. (2011). Transient Receptor Potential Canonical Channels Are Essential for Chemotactic Migration of Human Malignant Gliomas. J. Cell Physiol. 226 (7), 1879–1888. 10.1161/CIRCULATIONAHA.110.956839 21506118 PMC3841760

[B21] Bos J. L. (1989). Ras Oncogenes in Human Cancer: A Review. Cancer Res. 49 (17), 4682–4689. 2547513

[B22] Brossa A. Buono L. Fallo S. Fiorio Pla A. Munaron L. Bussolati B. (2019). Alternative Strategies to Inhibit Tumor Vascularization. Int. J. Mol. Sci. 20 (24), 6180. 10.3390/ijms20246180 PMC6940973 31817884

[B23] Bryan B. A. D’Amore P. A. (2007). What Tangled Webs They Weave: Rho-GTPase Control of Angiogenesis. Cell. Mol. Life Sci. 64 (16), 2053–2065. 10.1007/s00018-007-7008-z 17530172 PMC11138424

[B24] Cáceres M. Ortiz L. Recabarren T. Romero A. Colombo A. Leiva-Salcedo E. (2015). TRPM4 Is a Novel Component of the Adhesome Required for Focal Adhesion Disassembly, Migration and Contractility. PloS One 10 (6), 1–23. 10.1371/journal.pone.0130540 PMC4482413 26110647

[B25] Cai N. Lou L. Al-Saadi N. Tetteh S. Runnels L. W. (2018). The Kinase Activity of the Channel-Kinase Protein TRPM7 Regulates Stability and Localization of the TRPM7 Channel in Polarized Epithelial Cells. J. Biol. Chem. 293 (29), 11491–11504. 10.1074/jbc.RA118.001925 29866880 PMC6065181

[B26] Canales J. Morales D. Blanco C. Rivas J. Diaz N. Angelopoulos I. (2019). A Tr(i)p to Cell Migration: New Roles of Trp Channels in Mechanotransduction and Cancer. Front. Physiol. 10:757 (JUN). 10.3389/fphys.2019.00757 31275168 PMC6591513

[B27] Cao R. Meng Z. Liu T. Wang G. Qian G. Cao T. (2016). Decreased TRPM7 Inhibits Activities and Induces Apoptosis of Bladder Cancer Cells via ERK1 / 2 Pathway. Oncotarget 7 (45), 72941–72960. 10.18632/oncotarget.12146 27662662 PMC5341955

[B28] Cappelli H. C. Kanugula A. K. Adapala R. K. Amin V. Sharma P. Midha P. (2019). Mechanosensitive TRPV4 Channels Stabilize VE-Cadherin Junctions to Regulate Tumor Vascular Integrity and Metastasis. Cancer Lett. 442, 15–20. 10.1016/j.canlet.2018.07.042 30401632 PMC6924277

[B29] Caprodossi S. Lucciarini R. Amantini C. Nabissi M. Canesin G. Ballarini P. (2008). Transient Receptor Potential Vanilloid Type 2 (TRPV2) Expression in Normal Urothelium and in Urothelial Carcinoma of Human Bladder: Correlation with the Pathologic Stage. Eur. Urol. 54 (3), 612–620. 10.1016/j.eururo.2007.10.016 17977643

[B30] Carmeliet P. Jain R. K. (2000). Angiogenesis in Cancer and Other Diseases. Nature 407 (6801), 249–257. 10.1038/35025220 11001068

[B31] Carmeliet P. Jain R. K. (2011). Principles and Mechanisms of Vessel Normalization for Cancer and Other Angiogenic Diseases. Nat. Rev. Drug Discovery 10 (6), 417–427. 10.1038/ndr3455 21629292

[B32] Carmona G. Go S. Orlandi A. Ba T. Jugold M. Kiessling F. (2009). Role of the Small GTPase Rap1 for Integrin Activity Regulation in Endothelial Cells and Angiogenesis. Blood 113 (2), 488–497. 10.1182/blood-2008-02-138438 18805968

[B33] Chen J. P. Wang J. Luan Y. Wang C. X. Li W. H. Zhang J. B. (2015). TRPM7 Promotes the Metastatic Process in Human Nasopharyngeal Carcinoma. Cancer Lett. 356 (2), 483–490. 10.1016/j.canlet.2014.09.032 25304381

[B34] Chen L. Cao R. Wang G. Yuan L. Qian G. (2017). Downregulation of TRPM7 Suppressed Migration and Invasion by Regulating Epithelial – Mesenchymal Transition in Prostate Cancer Cells. Med. Oncol. 34 (7), 1–11. 10.1007/s12032-017-0987-1 28573641

[B35] Chen Z. Zhu Y. Dong Y. Zhang P. Han X. Jin J. (2017). Overexpression of TrpC5 Promotes Tumor Metastasis via the HIF-1α-Twist Signaling Pathway in Colon Cancer. Clin. Sci. 131 (19), 2439–2450. 10.1042/CS20171069 28864720

[B36] Cherfils J. Zeghouf M. (2013). Regulation of Small GTPases by GEFs, GAPs, and GDIs. Physiol. Rev. 93 (1), 269–309. 10.1152/physrev.00003.2012 23303910

[B37] Chigurupati S. Venkataraman R. Barrera D. Naganathan A. Madan M. Paul L. (2010). Receptor Channel TRPC6 Is a Key Mediator of Notch-Driven Glioblastoma Growth and Invasiveness. Cancer Res. 6 (11), 418–428. 10.1158/0008-5472.CAN-09-2654 20028870

[B38] Chrzanowska-wodnicka M. Kraus A. E. Gale D. Ii G. C.W. Vansluys J. (2008). Defective Angiogenesis, Endothelial Migration, Proliferation, and MAPK Signaling in Rap1b-Deficient Mice. Blood 111 (5), 2647–2656. 10.1182/blood-2007-08-109710 17993608 PMC2254536

[B39] Chrzanowska-Wodnicka M. (2010). Regulation of Angiogenesis by a Small GTPase Rap1. Vasc. Pharmacol. 53 (1–2), 1–10. 10.1016/j.vph.2010.03.003 20302970

[B40] Chung H. K. Rathor N. Wang S. R. Wang J. Y. Rao J. N. (2015). RhoA Enhances Store-Operated Ca2+ Entry and Intestinal Epithelial Restitution by Interacting with TRPC1 after Wounding. Am. J. Physiol. - Gastrointest. Liver Physiol. 309 (9), G759–G767. 10.1152/ajpgi.00185.2015 26336927 PMC4628965

[B41] Clark K. Middelbeek J. Lasonder E. Dulyaninova N. G. Morrice A. Ryazanov A. G. (2008). TRPM7 Regulates Myosin IIA Filament Stability and Protein Localization by Heavy Chain Phosphorylation. J. Mol. Biol. 378 (4), 790–803. 10.1016/j.jmb.2008.02.057 18394644 PMC4541798

[B42] Clayton N. S. Ridley A. J. (2020). Targeting Rho GTPase Signaling Networks in Cancer. Front. Cell Dev. Biol. 8:222. 10.3389/fcell.2020.00222 32309283 PMC7145979

[B43] Correll R. N. Pang C. Niedowicz D. M. Finlin B. S. Andres D. A. (2008). The RGK Family of GTP-Binding Proteins: Regulators of Voltage-Dependent Calcium Channels and Cytoskeleton Remodeling. Cell. Signal. 20 (2), 292–300. 10.1016/j.cellsig.2007.10.028 18042346 PMC2254326

[B44] Cullen P. J. Lockyer P. J. (2002). Integration of Calcium and Ras Signalling. Nat. Rev. Mol. Cell Biol. 3 (5), 339–348. 10.1038/nrm808 11988768

[B45] Davis F. M. Azimi I. Faville R. A. Peters A. A. Jalink K. Putney J. W. Jr. (2014). Induction of Epithelial – Mesenchymal Transition (EMT ) in Breast Cancer Cells Is Calcium Signal Dependent. Oncogene 33 (18), 2307–2316. 10.1038/onc.2013.187 23686305 PMC3917976

[B46] Desai B. N. Krapivinsky G. Navarro B. Krapivinsky L. Carter B. C. Febvay S. (2012). Cleavage of TRPM7 Releases the Kinase Domain from the Ion Channel and Regulates Its Participation in Fas-Induced Apoptosis. Dev. Cell 22 (6), 1149–1162. 10.1016/j.devcel.2012.04.006 22698280 PMC3397829

[B47] Dhennin-duthille I. Gautier M. Faouzi M. Brevet M. Vaudry D. Ahidouch A. (2011). Cellular Physiology and Biochemistr y Biochemistry High Expression of Transient Receptor Potential Channels in Human Breast Cancer Epithelial Cells and Tissues: Correlation with Pathological Parameters. Cell Physiol. Biochem. 28, 813–822. 22178934 10.1159/000335795

[B48] Dong H. Shim K. N. Li J. M. J. Estrema C. Ornelas T. A. Nguyen F. (2010). Molecular Mechanisms Underlying Ca2+-Mediated Motility of Human Pancreatic Duct Cells. Am. J. Physiol. - Cell Physiol. 299 (6), 1493–1503. 10.1152/ajpcell.00242.2010 PMC3006328 20861471

[B49] Elbaz M. Ahirwar D. Xiaoli Z. Zhou X. Lustberg M. Nasser M. W. (2018). TRPV2 Is a Novel Biomarker and Therapeutic Target in Triple Negative Breast Cancer. Oncotarget 9 (71), 33459–33470. 10.18632/oncotarget.9663 30323891 PMC6173360

[B50] Fabian A. Fortmann T. Dieterich P. Riethmüller C. Schön P. Mally S. (2008). TRPC1 Channels Regulate Directionality of Migrating Cells. Pflugers Archiv. Eur. J. Physiol. 457 (2), 475–484. 10.1007/s00424-008-0515-4 18542994

[B51] Faouzi M. Kilch T. Horgen F. D. Fleig A. Penner R. (2017). The TRPM7 Channel Kinase Regulates Store-Operated Calcium Entry. J. Physiol. 595 (10), 3165–3180. 10.1113/JP274006 28130783 PMC5430208

[B52] Fels B. Bulk E. Pethő Z. Schwab A. (2018). The Role of TRP Channels in the Metastatic Cascade. Pharmaceuticals 11 (2), 48. 10.3390/ph11020048 PMC6027473 29772843

[B53] Fiorio Pla A. F. Gkika D. (2013). Emerging Role of TRP Channels in Cell Migration : From Tumor Vascularization to Metastasis. Front. Physiol. 4, 1–12. 10.3389/fphys.2013.00311 24204345 PMC3817680

[B54] Fiorio Pla A. Ong H. L. Cheng K. T. Brossa A. Bussolati B. Lockwich T. (2012). TRPV4 Mediates Tumor-Derived Endothelial Cell Migration via Arachidonic Acid-Activated Actin Remodeling. Oncogene 31 (2), 200–212. 10.1016/j.physbeh.2017.03.040 21685934 PMC5934994

[B55] Fuessel S. Sickert D. Meye A. Klenk U. Schmidt U. Schmitz M. (2003). Multiple Tumor Marker Analyses (PSA, HK2, PSCA, Trp-P8) in Primary Prostate Cancers Using Quantitative RT-PCR. Int. J. Oncol. 23 (1), 221–228. 10.3892/ijo.23.1.221 12792797

[B56] Gao G. Wang W. Tadagavadi R. K. Briley N. E. Love M. II Miller B. A. (2014). TRPM2 Mediates Ischemic Kidney Injury and Oxidant Stress through RAC1. J. Clin. Invest. 124 (11), 4989–5001. 10.1172/JCI76042.us 25295536 PMC4347231

[B57] Genova T. Grolez G. P. Camillo C. Bernardini M. Bokhobza A. Richard E. (2017). TRPM8 Inhibits Endothelial Cell Migration via a Nonchannel Function by Trapping the Small GTPase Rap1. J. Cell Biol. 216 (7), 2107–2130. 10.1083/jcb.201506024 28550110 PMC5496606

[B58] Ghosh K. Thodeti C. K. Dudley A. C. Mammoto A. Klagsbrun M. Ingber D. E. (2008). Tumor-Derived Endothelial Cells Exhibit Aberrant Rho-Mediated Mechanosensing and Abnormal Angiogenesis in Vitro. Proc. Natl. Acad. Sci. U. S. A. 105 (32), 11305–11310. 10.1073/pnas.0800835105 18685096 PMC2516246

[B59] Gkika D. Prevarskaya N. (2011). TRP Channels in Prostate Cancer: The Good, the Bad and the Ugly? Asian J. Androl. 13 (5), 673–676. 10.1038/aja.2011.18 21623387 PMC3739589

[B60] Greka A. Navarro B. Oancea E. Duggan A. Clapham D. E. (2003). TRPC5 Is a Regulator of Hippocampal Neurite Length and Growth Cone Morphology. Nat. Neurosci. 6 (8), 837–845. 10.1038/nn1092 12858178

[B61] Guéguinou M. Harnois T. Crottes D. Uguen A. Deliot N. Gambade A. (2016). SK3/TRPC1/Orai1 Complex Regulates SOCE-Dependent Colon Cancer Cell Migration: A Novel Opportunity to Modulate Anti- EGFR MAb Action by the Alkyl-Lipid Ohmline. Oncotarget 7 (24), 36168–36184. 10.18632/oncotarget.8786 27102434 PMC5094991

[B62] Guilbert A. Dhennin-Duthille I. Hiani Y. E. L. Haren N. Khorsi H. Sevestre H. (2008). Expression of TRPC6 Channels in Human Epithelial Breast Cancer Cells. BMC Cancer 8, 1–11. 10.1186/1471-2407-8-125 18452628 PMC2409351

[B63] Guilbert A. Gautier M. Dhennin-Duthille I. Rybarczyk P. Sahni J. Sevestre H. (2013). Transient Receptor Potential Melastatin 7 Is Involved in Oestrogen Receptor-Negative Metastatic Breast Cancer Cells Migration through Its Kinase Domain. Eur. J. Cancer 49 (17), 3694–3707. 10.1016/j.ejca.2013.07.008 23910495

[B64] Haws H. J. Mcneil M. A. Hansen M. D.H. (2016). Control of Cell Mechanics by RhoA and Calcium Fl Uxes during Epithelial Scattering. Tissue Barriers 4 (3), 1–155. 10.1080/21688370.2016.1187326 PMC4993584 27583192

[B65] Hicks K. Neil R. G.O. Dubinsky W. S. Brown R. C. Hicks K. O Neil R. G. (2010). TRPC-Mediated Actin-Myosin Contraction Is Critical for BBB Disruption Following Hypoxic Stress. Am. J. Physiol. Cell Physiol. 298, 1583–1593. 10.1152/ajpcell.00458.2009 PMC2889642 20164382

[B66] Ho H.-h. Chang C.-s. Ho W.-c. Liao S.-y. Wu C.-h. (2010). Anti-Metastasis Effects of Gallic Acid on Gastric Cancer Cells Involves Inhibition of NF- j B Activity and Downregulation of PI3K / AKT / Small GTPase Signals. Food Chem. Toxicol. 48 (8–9), 2508–2516. 10.1016/j.fct.2010.06.024 20600540

[B67] Hobbs G. A. Der C. J. Rossman K. L. (2016). RAS Isoforms and Mutations in Cancer at a Glance. J. Cell Sci. 129 (7), 1287–1292. 10.1242/jcs.182873 26985062 PMC4869631

[B68] Holzmann C. Kappel S. Kilch T. Jochum M. M. Urban S. K. Jung V. (2015). Transient Receptor Potential Melastatin 4 Channel Contributes to Migration of Androgen-Insensitive Prostate Cancer Cells. Oncotarget 6 (39), 41783–41793. 10.18632/oncotarget.6157 26496025 PMC4747188

[B69] Huang Y. W. Chang S. J. Harn H. II Huang H. T. Lin H. H. Shen M. R. (2015). Mechanosensitive Store-Operated Calcium Entry Regulates the Formation of Cell Polarity. J. Cell. Physiol. 230 (9), 2086–2097. 10.1002/jcp.24936 25639747

[B70] Iamshanova O. Pla A. F. Prevarskaya N. (2017). Molecular Mechanisms of Tumour Invasion: Regulation by Calcium Signals. J. Physiol. 595 (10), 3063–3075. 10.1113/JP272844 28304082 PMC5430231

[B71] Iftinca M. Hamid J. Chen L. Varela D. Tadayonnejad R. Altier C. (2007). Regulation of T-Type Calcium Channels by Rho-Associated Kinase. Nat. Neurosci. 10 (7), 854–860. 10.1038/nn1921 17558400

[B72] Jabłońska-trypuć A. Matejczyk M. Rosochacki S. (2016). Matrix Metalloproteinases (MMPs ), the Main Extracellular Matrix (ECM ) Enzymes in Collagen Degradation, as a Target for Anticancer Drugs Enzymes in Collagen Degradation, as a Target for Anticancer Drugs. J. Enzyme Inhib. Med. Chem. 31 (sup1), 177–183. 10.3109/14756366.2016.1161620 27028474

[B73] Jacob A. Jing J. Lee J. Schedin P. Gilbert S. M. Peden A. A. (2013). Rab40b Regulates Trafficking of MMP2 and MMP9 during Invadopodia Formation and Invasion of Breast Cancer Cells. J. Cell Sci. 126 (Pt20), 4647–4658. 10.1242/jcs.126573 23902685 PMC3795337

[B74] Jiang L. H. Gamper N. Beech D. J. (2011). Properties and Therapeutic Potential of Transient Receptor Potential Channels with Putative Roles in Adversity: Focus on TRPC5, TRPM2 and TRPA1. Curr. Drug Targets 12 (5), 724–736. 10.2174/138945011795378568 21291387 PMC3267159

[B75] Kim J. G. Islam R. Cho J. Y. Jeong H. Cap K. C. Park Y. (2018). Regulation of RhoA GTPase and Various Transcription Factors in the RhoA Pathway. J. Cell. Physiol. 233 (9), 6381–6392. 10.1002/jcp.26487 29377108 PMC13482483

[B76] Koike T. Kuzuya M. Asai T. Kanda S. Cheng X. W. (2000). Activation of MMP-2 by Clostridium Difficile Toxin B in Bovine Smooth Muscle Cells. Biochem. Biophys. Res. Commun. 46, 43–46. 10.1006/bbrc.2000.3630 11027636

[B77] Koopman W. J. H. Bosch R. R. Van Emst-De Vries S. E. Spaargaren M. De Pont Jan Joep H. H. M. Willems P. H. G. M. (2003). R-Ras Alters Ca2+ Homeostasis by Increasing the Ca2+ Leak across the Endoplasmic Reticular Membrane. J. Biol. Chem. 278 (16), 13672–13679. 10.1074/jbc.M211256200 12586830

[B78] Laragione T. Cheng K. F. Tanner M. R. He M. Beeton C. Al-Abed Y. (2015). THE CATION CHANNEL TRPV2 IS A NEW SUPPRESSOR OF ARTHRITIS SEVERITY, JOINT DAMAGE AND SYNOVIAL FIBROBLAST INVASION. Clin. Immunol. 158 (2), 183–192. 10.1016/j.clim.2015.04.001 25869297 PMC4617367

[B79] Laragione T. Harris C. Gulko P. S. (2019). TRPV2 Suppresses Rac1 and RhoA Activation and Invasion in Rheumatoid Arthritis Fibroblast-like Synoviocytes. Int. Immunopharmacol. 70, 268–273. 10.1016/j.intimp.2019.02.051 30851707

[B80] Lastraioli E. Iorio J. Arcangeli A. (2015). Ion Channel Expression as Promising Cancer Biomarker. Biochim. Biophys. Acta - Biomembr. 1848 (10), 2685–2702. 10.1016/j.bbamem.2014.12.016 25542783

[B81] Lee W. H. Choong L. Y. Mon N. N. Lu S. Lin Q. Pang B. (2016). “TRPV4 Regulates Breast Cancer Cell Extravasation, Stiffness and Actin Cortex. Sci. Rep. 6 (May), 1–16. 10.1038/srep27903 27291497 PMC4904279

[B82] Lee W. H. Choong L. Y. Jin T. H. Mon N. N. Chong S. Liew C. S. (2017). TRPV4 Plays a Role in Breast Cancer Cell Migration via Ca2+-Dependent Activation of AKT and Downregulation of E-Cadherin Cell Cortex Protein. Oncogenesis 6 (5), e338. 10.1038/oncsis.2017.39 28530703 PMC5523072

[B83] Lehen’kyi V. Prevarskaya N. (2011). “Oncogenic TRP Channels.” in Transient Receptor Potential Channels. Advances in Experimental Medicine and Biology. Ed. Islam M. (United States: Springer), 929–945. 10.1007/978-94-007-0265-3_48 21290334

[B84] Lepannetier S. Zanou N Yerna X. Emeriau N. Dufour I. Masquelier J. (2016). Sphingosine-1-Phosphate-Activated TRPC1 Channel Controls Chemotaxis of Glioblastoma Cells. Cell Calcium 60 (6), 373–383. 10.1016/j.ceca.2016.09.002 27638096

[B85] Li S. Balmain A. Counter C. M. (2018). A Model for RAS Mutation Patterns in Cancers: Finding the Sweet Spot. Nat. Rev. Cancer 18 (12), 767–777. 10.1038/s41568-018-0076-6 30420765

[B86] Litan A. Langhans S. A. (2015). Cancer as a Channelopathy: Ion Channels and Pumps in Tumor Development and Progression. Front. Cell. Neurosci. 9:86 (March). 10.3389/fncel.2015.00086 25852478 PMC4362317

[B87] Liu J. Chen Y. Shuai S. Ding D. (2014). TRPM8 Promotes Aggressiveness of Breast Cancer Cells by Regulating EMT via Activating AKT / GSK-3 β Pathway. Tumour Biol. 35 (9), 8969–8977. 10.1007/s13277-014-2077-8 24903376

[B88] Liu K. Xu S.-h. Chen Z. Zeng Q.-x. Li Z.-j. Chen Z.-m. (2018). TRPM7 Overexpression Enhances the Cancer Stem Cell-like and Metastatic Phenotypes of Lung Cancer through Modulation of the Hsp90 α / UPA / MMP2 Signaling Pathway. BMC Cancer 18 (1), 1167. 10.1186/s12885-018-5050-x 30477473 PMC6258145

[B89] Liu L. Wu N. Wang Y. Zhang X. Xia B. Tang J. (2019). TRPM7 Promotes the Epithelial – Mesenchymal Transition in Ovarian Cancer through the Calcium-Related PI3K / AKT Oncogenic Signaling. J. Exp. Clin. Cancer Res. 38 (1), 106–121. 10.1186/s13046-019-1061-y 30819230 PMC6396458

[B90] Llense F. Etienne-Manneville S. (2015). “Front-to-Rear Polarity in Migrating Cells.” in Cell Polarity 1. Ed. Klaus E. (United States: Springer), 115–146. 10.1007/978-3-319-14463-4_5

[B91] Luo Y. Wu J.-y. Lu M.-h. Shi Z. Na N. Di J.-m. (2016). Carvacrol Alleviates Prostate Cancer Cell Proliferation, Migration, and Invasion through Regulation of PI3K / Akt and MAPK Signaling Pathways. Oxid. Med. Cell Longev. 2016, 1469693. 10.1155/2016/1469693 27803760 PMC5075627

[B92] Mehta D. Ahmmed G. U. Paria B. C. Holinstat M. Voyno-Yasenetskaya T. Tiruppathi C. (2003). RhoA Interaction with Inositol 1,4,5-Trisphosphate Receptor and Transient Receptor Potential Channel-1 Regulates Ca2+ Entry: Role in Signaling Increased Endothelial Permeability. J. Biol. Chem. 278 (35), 33492–33500. 10.1074/jbc.M302401200 12766172

[B93] Meng X. Cai C. Wu J. Cai S. Ye C. Chen H. (2013). TRPM7 Mediates Breast Cancer Cell Migration and Invasion through the MAPK Pathway. Cancer Lett. 333 (1), 96–102. 10.1016/j.canlet.2013.01.031 23353055

[B94] Middelbeek J. Kuipers A. J. Henneman L. Visser D. Eidhof I. Van Horssen R. (2012). TRPM7 Is Required for Breast Tumor Cell Metastasis. Cancer Res. 72 (16), 4250–4261. 10.1158/0008-5472.CAN-11-3863 22871386

[B95] Mishra A. K. Lambright D. G. (2016). Small GTPases and Their GAPs. Biopolymers 105 (8), 431–448. 10.1002/bip.22833 26972107 PMC5439442

[B96] Moissoglu K. Schwartz M. A. (2014). Spatial and Temporal Control of Rho GTPase Functions. Cell. Logistics 4 (2), e943618. 10.4161/21592780.2014.943618 PMC4279778 25610718

[B97] Monet M. Lehen V. Gackiere F. Firlej V. Vandenberghe M. Roudbaraki M. (2010). Role of Cationic Channel TRPV2 in Promoting Prostate Cancer Migration and Progression to Androgen Resistance. Cancer Res. 70 (3), 1225–1236. 10.1158/0008-5472.CAN-09-2205 20103638

[B98] Monteith G. R. Prevarskaya N. Roberts-Thomson S. J. (2017). The Calcium–cancer Signalling Nexus. Nat. Rev. Cancer 17 (6), 367–380. 10.1038/nrc.2017.18 28386091

[B99] Mrkonjić S. Garcia-Elias A. Pardo-Pastor C. Bazellières E. Trepat X. Vriens J. (2015). TRPV4 Participates in the Establishment of Trailing Adhesions and Directional Persistence of Migrating Cells. Pflugers Archiv. Eur. J. Physiol. 467 (10), 2107–2119. 10.1007/s00424-014-1679-8 25559845

[B100] Nagasawa M. Kojima I. (2015). Translocation of TRPV2 Channel Induced by Focal Administration of Mechanical Stress. Physiol. Rep. 3, 1–12. 10.14814/phy2.12296 PMC4393204 25677550

[B101] Narayan G. Bourdon V. Chaganti S. Arias-Pulido H. Nandula S. V. Rao P. H. (2007). Gene Dosage Alterations Revealed by CDNA Microarray Analysis in Cervical Cancer: Identification of Candidate Amplified and Overexpressed Genes. Genes Chromosomes Cancer 46 (4), 373–384. 10.1002/gcc.20418 17243165

[B102] Negri S. Faris P. Berra-Romani R. Guerra G. Moccia F. (2020). Endothelial Transient Receptor Potential Channels and Vascular Remodeling: Extracellular Ca2 + Entry for Angiogenesis, Arteriogenesis and Vasculogenesis. Front. Physiol. 10, 1618 (January). 10.3389/fphys.2019.01618 32038296 PMC6985578

[B103] Nilius B. Owsianik G. (2011). The Transient Receptor Potential Family of Ion Channels. Genome Biol. 12 (3), 218. 10.1186/gb-2011-12-3-218 21401968 PMC3129667

[B104] Okamoto Y. Ohkubo T. Ikebe T. Yamazaki J. U. N. (2012). Blockade of TRPM8 Activity Reduces the Invasion Potential of Oral Squamous Carcinoma Cell Lines. Int. J. Oncol. 40 (5), 1431–1440. 10.3892/ijo.2012.1340 22267123

[B105] Oulidi A. Bokhobza A. Gkika D. Vanden Abeele F. Lehen V. Houcine Ouafik L. (2013). TRPV2 Mediates Adrenomedullin Stimulation of Prostate and Urothelial Cancer Cell Adhesion, Migration and Invasion. PLoS One 8 (5), 1–7. 10.1371/journal.pone.0064885 PMC3669125 23741410

[B106] Ou-yang Q. Li B. Xu M. Liang H. (2018). TRPV4 Promotes the Migration and Invasion of Glioma Cells via AKT/Rac1 Signaling. Biochem. Biophys. Res. Commun. 503 (2), 876–881. 10.1016/j.bbrc.2018.06.090 29928875

[B107] Prior I. A. Lewis P. D. Mattos C. (2012). A Comprehensive Survey of Ras Mutations in Cancer. Cancer Res. 72 (10), 2457–2467. 10.1158/0008-5472.CAN-11-2612.A 22589270 PMC3354961

[B108] Rampino T. Gregorini M. Guidetti C. Broggini M. Marchini S. Bonomi R. (2007). KCNA1 and TRPC6 Ion Channels and NHE1 Exchanger Operate the Biological Outcome of HGF / Scatter Factor in Renal Tubular Cells. Growth Factors 25, 382–391. 10.1080/08977190801892184 18365869

[B109] Ridley A. J. (2001). Rho Family Proteins : Coordinating Cell Responses. Growth Factors 11 (12), 471–477. 10.1016/s0962-8924(01)02153-5 11719051

[B110] Roche J. (2018). The Epithelial-to-Mesenchymal Transition in Cancer. Cancers 10 (3), 10–13. 10.3390/cancers10020052 PMC5836084 29462906

[B111] Rybarczyk P. Gautier M. Hague F. Dhennin-Duthille I. Chatelain D. Kerr-Conte J. (2012). Transient Receptor Potential Melastatin-Related 7 Channel Is Overexpressed in Human Pancreatic Ductal Adenocarcinomas and Regulates Human Pancreatic Cancer Cell Migration. Int. J. Cancer 131 (6), 851–861. 10.1002/ijc.27487 22323115

[B112] Rybarczyk P. Vanlaeys A. Brassart B. Dhennin-duthille I. Chatelain D. Sevestre H. (2017). The Transient Receptor Potential Melastatin 7 Channel Regulates Pancreatic Cancer Cell Invasion through the Hsp90 α / UPA / MMP2. Neoplasia 19 (4), 288–300. 10.1016/j.neo.2017.01.004 28284058 PMC5345960

[B113] Sahai E. Marshall C. J. (2002). RHO - GTPases and Cancer. Nat. Rev. Cancer 2 (2), 133–142. 10.1038/nrc725 12635176

[B114] Santibáñez J. F. Kocic J. Fabra A. Cano A. Quintanilla M. (2010). Rac1 Modulates TGF- b 1-Mediated Epithelial Cell Plasticity and MMP9 Production in Transformed Keratinocytes. FEBS Lett. 584 (11), 2305–2310. 10.1016/j.febslet.2010.03.042 20353788

[B115] Scarpellino S. Munaron L. Cantelmo A. R. Fiorio Pla A. (2020). Calcium-Permeable Channels in Tumor Vascularization: Peculiar Sensors of Microenvironmental Chemical and Physical Cues. Rev. Physiol. Biochem. Pharmacol. 10.1007/112_2020_32 32809072

[B116] Seiz J. R. Klinke J. Scharlibbe L. Lohfink D. Heipel M. (2020). Different Signaling and Functionality of Rac1 and Rac1b in the Progression of Lung Adenocarcinoma. Biol. Chem. 401 (4), 517–531. 10.1515/hsz-2019-0329 31811797

[B117] Shapovalov G. Ritaine A. Skryma R. Prevarskaya N. (2016). Role of TRP Ion Channels in Cancer and Tumorigenesis. Semin. Immunopathol. 38 (3), 357–369. 10.1007/s00281-015-0525-1 26842901

[B118] Shields J. M. Pruitt K. Shaub A. Der C. J. (2000). Understanding Ras : ‘ It Ain ‘ t over ‘ Til It ‘ s over ‘. Trends Cell Biol. 10 (4), 147–154. 10.1016/s0962-8924(00)01740-2 10740269

[B119] Simon A. R. Vikis H. G. Steward S. Fanburg B. L. Cochran B. H. Guan K.-L. (2000). Regulation of STAT3 by Direct Binding to the Racl GTPase. Science 290 (5489), 144–147. 10.1126/science.290.5489.144 11021801

[B120] Singh J. Manickam P. Shmoish M. Natik S. Denyer G. Handelsman D. (2006). Annotation of Androgen Dependence to Human Prostate Cancer-Associated Genes by Microarray Analysis of Mouse Prostate. Cancer Lett. 237 (2), 298–304. 10.1016/j.canlet.2005.06.008 16024171

[B121] Singh I. Knezevic N. Ahmmed G. U. Kini V. Malik A. B. Mehta D. (2007). Gαq -TRPC6-Mediated Ca 2+ Entry Induces RhoA Activation and Resultant Endothelial Cell Shape Change in Response to Thrombin. J. Biol. Chem. 282 (11), 7833–7843. 10.1074/jbc.M608288200 17197445

[B122] Sokabe T. Tominaga M. (2010). The TRPV4 Cation Channel. Communicative Integr. Biol. 3 (6), 619–621. 10.4161/cib.3.6.13461 PMC3038082 21331258

[B123] Stenmark H. (2009). Rab GTPases as Coordinators of Vesicle Traffic. Nat. Rev. Mol. Cell Biol. 10 (8), 513–525. 10.1038/nrm2728 19603039

[B124] Su L.-t. Liu W. Chen H.-c. An O. G. A.-p. Habas R. Runnels L. W. (2011). TRPM7 Regulates Polarized Cell Movements. Biochem. J. 434 (3), 513–521. 10.1042/BJ20101678 21208190 PMC3507444

[B125] Sumoza-Toledo A. Espinoza-Gabriel M. II Montiel-Condado D. (2016). Evaluación Del Canal TRPM2 Como Biomarcador En Cáncer de Mama Mediante El Análisis de Bases de Datos Públicos. Boletin Medico Del Hosp. Infantil Mexico 73 (6), 397–404. 10.1016/j.bmhimx.2016.10.001 29421284

[B126] Sun J. Yang T. Wang P. Ma S. Zhu Z. Pu Y. (2014). Activation of Cold-Sensing Transient Receptor Potential Melastatin Subtype 8 Antagonizes Vasoconstriction and Hypertension through Attenuating RhoA/Rho Kinase Pathway. Hypertension 63 (6), 1354–1363. 10.1161/HYPERTENSIONAHA.113.02573 24637663

[B127] Svensmark J. H. Brakebusch C. (2019). Rho GTPases in Cancer: Friend or Foe? Oncogene 38 (50), 7447–7456. 10.1038/s41388-019-0963-7 31427738

[B128] Thodeti C. K. Matthews B. Ravi A. Mammoto A. Ghosh K. Bracha A. L. (2009). TRPV4 Channels Mediate Cyclic Strain-Induced Endothelial Cell Reorientation through Integrin-to-Integrin Signaling. Circ. Res. 104 (9), 1123–1130. 10.1161/CIRCRESAHA.108.192930 19359599 PMC2754067

[B129] Thodeti C. K. Paruchuri S. Meszaros J. G. (2013). A TRP to Cardiac Fibroblast Differentiation. Channels 7 (3), 211–214. 10.4161/chan.24328 23511028 PMC3710348

[B130] Thoppil R. J. Cappelli H. C. Adapala R. K. Kanugula A. K. Paruchuri S. Thodeti C. K. (2016). TRPV4 Channels Regulate Tumor Angiogenesis via Modulation of Rho/Rho Kinase Pathway. Oncotarget 7 (18), 25849–25861. 10.18632/oncotarget.8405 27029071 PMC5041949

[B131] Tian D. Jacobo S. M.P. Billing D. Rozkalne A. Gage S. D. Anagnostou T. (2010). Antagonistic Regulation Od Actin Dynamics and Cell Motility by TRPC5 and TRPC6 Channels. Sci. Signal. 3 (145), 1–25. 10.1126/scisignal.2001200.Antagonistic PMC3071756 20978238

[B132] Tomasek J. J. Vaughan M. B. Kropp B. P. Gabbiani G. Martin M. D. Hinz B. (2006). Contraction of Myofibroblasts in Granulation Tissue Is Dependent on Rho / Rho Kinase / Myosin Light Chain Phosphatase Activity. Wound Repair Regen. 14 (3), 313–320. 10.1111/j.1743-6109.2006.00126.x 16808810

[B133] Tsavaler L. Shapero M. H. Morkowski S. Laus R. (2001). Trp-P8, a Novel Prostate-Specific Gene, Is up-Regulated in Prostate Cancer and Other Malignancies and Shares High Homology with Transient Receptor Potential Calcium Channel Proteins. Cancer Res. 61 (9), 3760–3769. 11325849

[B134] Ungefroren H. Witte D. Lehnert H. (2018). The Role of Small GTPases of the Rho/Rac Family in TGF-β-Induced EMT and Cell Motility in Cancer. Dev. Dynamics 247 (3), 451–461. 10.1002/dvdy.24505 28390160

[B135] Vennekens R. Nilius B. (2007). “Insights into TRPM4 Function, Regulation and Physiological Role.” in Transient Receptor Potential (TRP) Channels. Handbook of Experimental Pharmacology. Eds. Flockerzi V. Nilius B. (United States: Springer), 269–285. 10.1007/978-3-540-34891-7_16 17217063

[B136] Vetter I. R. Wittinghofer A. (2001). The Guanine Nucleotide-Binding Switch in Three Dimensions. Science 294 (5545), 1299–1304. 10.1126/science.1062023 11701921

[B137] Vrenken K. S. Jalink K. van Leeuwen F. N. Middelbeek J. (2015). Beyond Ion-Conduction: Channel-Dependent and -Independent Roles of TRP Channels during Development and Tissue Homeostasis. Biochim. Biophys. Acta - Mol. Cell Res. 1863 (6), 1436–1446. 10.1016/j.bbamcr.2015.11.008 26585368

[B138] Vriens J. Janssens A. Prenen J. Nilius B. Wondergem R. (2004). TRPV Channels and Modulation by Hepatocyte Growth Factor / Scatter Factor in Human Hepatoblastoma (HepG2 ) Cells. Cell Calcium 36 (1), 19–28. 10.1016/j.ceca.2003.11.006 15126053

[B139] Wang Y. He H. Srivastava N. Vikarunnessa S. Chen Y. B. Jiang J. (2012). Plexins Are GTPase-Activating Proteins for Rap and Are Activated by Induced Dimerization. Sci. Signaling 5 (207), 1–25. 10.1126/scisignal.2002636 PMC3413289 22253263

[B140] Wei C. Wang X. Chen M. Ouyang K. Song L.-S. Cheng H. (2009). Calcium Flickers Steer Cell Migration. Nature 457 (7231), 901–905. 10.1038/nature07577.Calcium 19118385 PMC3505761

[B141] Xiao N. Jiang L. M. Ge B. Zhang T. Y. Zhao X. K. Zhou X. (2014). Over-Expression of TRPM8 Is Associated with Poor Prognosis in Urothelial Carcinoma of Bladder. Tumor Biol. 35 (11), 11499–11504. 10.1007/s13277-014-2480-1 25128062

[B142] Xie R. Xu J. Xiao Y. Wu J. Wan H. Tang B. (2017). Calcium Promotes Human Gastric Cancer via a Novel Coupling of Calcium-Sensing Receptor and TRPV4 Channel. Cancer Res. 77 (23), 6499–6512. 10.1158/0008-5472.CAN-17-0360 28951460

[B143] Xu S.-z. Muraki K. Zeng F. Li J. Sukumar P. Dedman A. M. (2006). A Sphingosine-1 – Phosphate – Activated Calcium Channel Controlling Vascular Smooth Muscle Cell Motility. Cell. Pharmacol. 98 (11), 1381–1389. 10.1161/01.RES.0000225284.36490.a2.A PMC2648505 16675717

[B144] Yang D. Kim J. (2020). Emerging Role of Transient Receptor Potential (TRP) Channels in Cancer Progression. BMB Rep. 53 (3), 125–132. 10.5483/BMBRep.2020.53.3.016 32172727 PMC7118349

[B145] Yang J. Liu W. Lu X. Fu Y. Li L. Luo Y. (2015). High Expression of Small GTPase Rab3D Promotes Cancer Progression and Metastasis. Oncotarget 6 (13), 11125–11138. 10.18632/oncotarget.3575 25823663 PMC4484444

[B146] Yarwood S. Cullen P. J. Kupzig S. (2006). The GAP1 Family of GTPase-Activating Proteins : Spatial and Temporal Regulators of Small GTPase Signalling. Biochem. Soc. Trans. 34 (5), 846–850. 10.1042/BST0340846 17052212

[B147] Yee N. S. Kazi A. A. Li Q. Yang Z. Berg A. Yee R. K. (2015). Aberrant Over-Expression of TRPM7 Ion Channels in Pancreatic Cancer: Required for Cancer Cell Invasion and Implicated in Tumor Growth and Metastasis. Biol. Open 4 (4), 507–514. 10.1242/bio.20137088 25770184 PMC4400593

[B148] Yue D. Wang Y. Xiao J. Y. Wang P. Ren C. S. (2009). Expression of TRPC6 in Benign and Malignant Human Prostate Tissues. Asian J. Androl. 11 (5), 541–547. 10.1038/aja.2009.53 19701218 PMC3735007

[B149] Zahra F. T. Sajib Md S. Ichiyama Y. Akwii R. G. Tullar P. E. Cobos C. (2019). Endothelial RhoA GTPase Is Essential for in Vitro Endothelial Functions but Dispensable for Physiological in Vivo Angiogenesis. Sci. Rep. 9 (1), 1–15. 10.1038/s41598-019-48053-z 31406143 PMC6690958

[B150] Zeng Z. Leng T. Feng X. Sun H. Inoue K. Zhu L. (2015). Silencing TRPM7 in Mouse Cortical Astrocytes Impairs Cell Proliferation and Migration via ERK and JNK Signaling Pathways. PloS One 10 (3), 1–19. 10.1371/journal.pone.0119912 PMC4370640 25799367

[B151] Zhang B. Zhang Y. Wang Z. X. Zheng Y. (2000). The Role of Mg2+ Cofactor in the Guanine Nucleotide Exchange and GTP Hydrolysis Reactions of Rho Family GTP-Binding Proteins. J. Biol. Chem. 275 (33), 25299–25307. 10.1074/jbc.M001027200 10843989

[B152] Zhang S. S. Wen J. Yang F. Cai X. L. Yang H. Luo K. J. (2013). High Expression of Transient Potential Receptor C6 Correlated with Poor Prognosis in Patients with Esophageal Squamous Cell Carcinoma. Med. Oncol. 30 (3), 607. 10.1007/s12032-013-0607-7 23686700

[B153] Zhao W. Xu H. (2016). High Expression of TRPM8 Predicts Poor Prognosis in Patients with Osteosarcoma. Oncol. Lett. 12 (2), 1373–1379. 10.3892/ol.2016.4764 27446440 PMC4950156

[B154] Zhou K. Zhang S. S. Yan Y. Zhao S. (2014). Overexpression of Transient Receptor Potential Vanilloid 2 Is Associated with Poor Prognosis in Patients with Esophageal Squamous Cell Carcinoma. Med. Oncol. 31 (7), 17. 10.1007/s12032-014-0017-5 24878697

[B155] Zhuge Y. Xu J. (2001). Rac1 Mediates Type I Collagen-Dependent MMP-2 Activation. J. Biol. Chem. 276 (19), 16248–16256. 10.10974/jbc.M01090200 11340084

[B156] Zoppoli P. Calice G. Laurino S. Ruggieri V. La Rocca F. La Torre G. (2019). TRPV2 Calcium Channel Gene Expression and Outcomes in Gastric Cancer Patients: A Clinically Relevant Association. J. Clin. Med. 8 (5):662. 10.3390/jcm8050662 PMC6572141 31083561

